# CQLHBA: Node Coverage Optimization Using Chaotic Quantum-Inspired Leader Honey Badger Algorithm

**DOI:** 10.3390/biomimetics10120850

**Published:** 2025-12-18

**Authors:** Xiaoliu Yang, Mengjian Zhang

**Affiliations:** 1 Department of Automation Engineering, Moutai Institute, Renhuai 564507, China; 2 School of Artificial Intelligence and Information Technology, Nanjing University of Chinese Medicine, Nanjing 210023, China

**Keywords:** honey badger algorithm, bio-inspired optimization, chaotic encoding, quantum rotation, wireless sensor network, node coverage

## Abstract

A key limitation of existing swarm intelligence (SI) algorithms for Node Coverage Optimization (NCO) is their inadequate solution accuracy. A novel chaotic quantum-inspired leader honey badger algorithm (CQLHBA) is proposed in this study. To enhance the performance of the basic HBA and better solve the numerical optimization and NCO problem, an adjustment strategy for parameter $\alpha_{1}$ to balance the optimization process of the follower position is used to improve the exploration ability. Moreover, the chaotic dynamic strategy, quantum rotation strategy, and Lévy flight strategy are employed to enhance the overall performance of the designed CQLHBA, especially for the exploitation ability of individuals. The performance of the proposed CQLHBA is verified using twenty-one benchmark functions and compared to that of other state-of-the-art (SOTA) SI algorithms, including the Honey Badger Algorithm (HBA), Chaotic Sea-Horse Optimizer (CSHO), Sine–Cosine Quantum Salp Swarm Algorithm (SCQSSA), Golden Jackal Optimization (GJO), Aquila Optimizer (AO), Butterfly Optimization Algorithm (BOA), Salp Swarm Algorithm (SSA), Grey Wolf Optimizer (GWO), and Randomised Particle Swarm Optimizer (RPSO). The experimental results demonstrate that the proposed CQLHBA exhibits superior performance, characterized by enhanced global search capability and robust stability. This advantage is further validated through its application to the NCO problem in wireless sensor networks (WSNs), where it achieves commendable outcomes in terms of both coverage rate and network connectivity, confirming its practical efficacy in real-world deployment scenarios.

## 1. Introduction

Meta-heuristic algorithms, inspired by natural phenomena, are indispensable tools for solving complex optimization problems in fields such as engineering and economic management. From established algorithms like the Genetic Algorithm (GA) [1,2] and Particle Swarm Optimization (PSO) [3,4,5] to newer ones like the Grey Wolf Optimizer (GWO) [6] and Whale Optimization Algorithm (WOA) [7], the field continues to evolve, with the Honey Badger Algorithm (HBA) being a recent example introduced by Hashim et al. [8]. A critical application area for these algorithms is node coverage optimization in wireless sensor networks (WSNs) [9,10], which is a complex combinatorial problem aimed at maximizing network lifetime and minimizing energy consumption or coverage blind spots. The strong global search capabilities of meta-heuristics make them a mainstream solution for this problem; numerous other improved meta-heuristic algorithms have achieved remarkable success for this task. However, its performance still needs to be further improved, especially by adopting HBA.

To address the inherent limitations of the standard HBA, numerous enhanced versions have been developed, centering mainly on three improvement pathways: hybridizing with other techniques, refining parameter adaptation mechanisms, and modifying population topological structures. For example, in the domain of hybrid strategies, Huang et al. [11] introduced a chaotic HBA variant (CHBA) leveraging Tent chaotic mapping to enrich initial population diversity. Meanwhile, Sheng et al. [12] designed a hybrid framework combining HBA with differential evolution (HHBADE), effectively balancing global exploratory behavior and local refinement capabilities. More recently, Guo et al. [13] proposed a multi-strategy HBA (MSHBA) incorporating cubic mapping for initialization, along with random search, elite tangent search, and differential mutation strategies, further enhancing search efficiency and robustness. Parameter optimization represents another critical research thread. Dixit et al. [14] integrated Lévy flight perturbations into HBA’s update process, enabling the algorithm to escape local optima more readily and improving convergence accuracy in complex multimodal landscapes. Beyond parameter control, altering population topology has also yielded promising outcomes. A notable example is the dynamic multi-population HBA (DMPHBA) proposed by Xu et al. [15], which employs a symbiotic mechanism-based strategy to facilitate information exchange among subpopulations, thus preserving diversity throughout the evolutionary process.The differences in algorithm strategies and optimization capabilities among HBA variants are shown in Table 1.

The optimization of node coverage is of paramount importance for enhancing the overall performance and functionality of wireless sensor network (WSN) operational environments. In recent years, swarm intelligence (SI) algorithms have made pivotal contributions to addressing the Node Coverage Optimization (NCO) challenge in WSNs. For instance, Yang et al. [17] tackled the sensor coverage problem using an enhanced Firefly Algorithm (FA), which simultaneously considers both target coverage requirements and the connectivity among network nodes. Additionally, two bio-inspired optimization techniques modeled after wolf behaviors have been applied to the NCO problem. These include an improved Grey Wolf Optimizer with a multi-strategy (IGWO-MS) algorithm [18] and a wolf pack algorithm (WPA) integrated with a coverage-oriented methodology [19]. More recently, Zhang et al. [20] developed a hybrid node coverage optimization approach and validated its performance across various simulated environments. Yu et al. [21] designed an adaptive learning Grey Wolf Optimizer (ALGWO) with a dynamic opposite learning strategy to address the NCO task. Chen et al. [16] introduced a hybrid butterfly–beluga whale optimization algorithm (HBBWOA) with a dynamic strategy for the 2D NCO issue. While these SI-driven methods have demonstrated considerable success in improving coverage rates and network efficiency, they still encounter difficulties in mitigating premature convergence and escaping local optima, particularly in complex or large-scale deployment scenarios. Consequently, the continued investigation and development of novel heuristic and meta-heuristic algorithms remain both essential and highly valuable for advancing the state of the art in WSN coverage optimization. Table 2 summarizes the shortcomings of existing SI algorithms for WSN’s NCO problem.

Aiming at the shortcomings of the HBA, a novel chaotic quantum-inspired leader honey badger algorithm (CQLHBA) is proposed with multi-strategies of honey badger behaviors with in-depth thinking. First, a nonlinear dynamic strategy, denoted as $\alpha_{1}$, is employed to regulate the behavior of follower honey badgers during the optimization process, enabling adaptive adjustments in response to evolving search conditions. Second, a chaotic dynamic strategy is introduced to enhance the exploratory capability of the leader during the digging phase, facilitating escape from local optima and promoting more thorough space exploitation. Furthermore, the integration of quantum rotation operations with Lévy flight mechanisms serves to dynamically modulate the search scope of individuals throughout the optimization process, thereby effectively balancing global exploration and local refinement. This synergistic combination significantly augments both the global search capacity and local convergence precision of the proposed CQLHBA. The main contributions are summarized as follows, given the above motivation for this study:

— According to honey badger behaviors in nature, a novel CQLHBA is proposed. A dual-strategy framework assigns chaotic and nonlinear dynamics to leaders and followers for specialized optimization. In addition, a hybrid search operator combines Lévy flight with quantum rotation, enhancing the exploration–precision balance.
— A rigorous comparative analysis on twenty-one CEC benchmark functions validates the superior performance of the CQLHBA over other advanced swarm intelligence algorithms.
— The proposed CQLHBA is applied to the formulated Node Coverage Optimization (NCO) problem for IoT-based WSNs and exhibits superior performance in a comparative analysis against several prominent SI methods.

## 2. Theory of Honey Badger Algorithm

The Honey Badger Algorithm is a swarm-based optimization method inspired by the natural foraging behavior of honey badgers. The mathematical representation of the system’s behavior is the optimization process inherent in the designed HBA. Three key phases of the HBA can be summarized as the initialization phase, digging phase and honey phase.

### 2.1. Initialization Phase

The problem under consideration is assumed to be defined within a D-dimensional search space. Accordingly, the initial positions for the entire honey badger population are generated at random, ensuring that the individuals are widely dispersed across the available solution domain. This random initialization is a standard and crucial procedure in population-based algorithms, as it promotes the initial exploration phase by sampling a diverse set of potential solutions, which is defined as   

(1) $$X_{i,j}=rand_{i,j}\cdot \left(ub_{i,j}-lb_{i,j}\right)+lb_{i,j}$$

where $X_{i,j}$ signifies the initial position of an individual honey badger. The index i=1,2,⋯,NP ranges over the population, while j=1,2,⋯,D ranges over the problem dimensions. $rand_{i,j}$ denotes a random number sampled from a uniform distribution in the interval (0,1). The search domain is constrained by $ub_{i,j}$ and $lb_{i,j}$, which represent the upper and lower bounds, respectively.

### 2.2. Digging Phase and Honey Phase

The position of the honey badger is updated using the Cardioid shape strategy in the digging phase (rand<0.5) and honey phase (rand≥0.5), which can be defined as

(2) $$X_{\mathrm{new}}=\left\{ \begin{array}{cc} X_{\mathrm{prey}}+F\times \Phi \times I\times X_{\mathrm{prey}} & \mathrm{if}\mathrm{rand}<0.5\\ \;\;+F\times r_{1}\times \alpha_{0}\times d_{i}\times \left|\cos\left(2\pi \cdot r_{2}\right)\times \left[1-\cos\left(2\pi \cdot r_{3}\right)\right]\right|,\\ X\mathrm{prey}+F\times r_{4}\times \alpha_{0}\times d_{i}, & \mathrm{if}\mathrm{rand}\geq 0.5 \end{array}\right.$$

where $X_{prey}$ denotes position of the best prey of the badger; $X_{new}$ is the solution of the updating individual; and Φ represents the capability to obtain food (Φ = 6 in this study). *I* denotes the smell intensity of the prey $X_{prey}$. $d_{i}$ measures the distance from the *i*-th honey badger to the prey. $r_{1}$ to $r_{4}$ are different random values in [0, 1]. $\alpha_{0}$ is the update density factor. The parameters of *I*, $d_{i}$, and $\alpha_{0}$ are, respectively, defined as

(3) $$I_{i}=rand\times \frac{{\left(X_{i}-X_{i+1}\right)}^{2}}{4\pi \cdot d_{i}^{2}}$$

(4) $$d_{i}=X_{prey}-X_{i}$$

(5) $$\alpha_{0}=C\times exp\left(\frac{-t}{T_{max}}\right)$$

where $X_{i}$ denotes the current solution, and $X_{i+1}$ indicates the (*i*+1)-th solution. *C* is a constant, which is set to 2 in this study. *t* is the current iteration, and $T_{max}$ denotes the max iterations. rand is a random number in (0, 1).

In addition, variable *F* defines the direction of the search process, which is defined as

(6) $$F=\{\begin{array}{cc} & 1,\;if\;rand\leq 0.5\\ & -1,\;else \end{array}$$

## 3. The Designed Chaotic Quantum Honey Badger Algorithm

In this study, to overcome the shortage of HBA falling into local optimum and use the improved method to optimize hyperparameters for the WSN node coverage optimization problem, we propose an adjustment strategy for parameter $\alpha_{1}$ to balance the optimization process of the follower position. Moreover, the chaotic dynamic strategy, quantum rotation strategy, and Lévy flight strategy are employed to enhance the overall performance of the designed CQLHBA. Three processes of the proposed CQLHBA are summarized as dynamic control mechanisms, exploration enhancements, and exploitation enhancements.

### 3.1. Dynamic Control Mechanisms

To enhance the performance between the exploration and exploitation of the follower, the adjustment strategy of parameter $\alpha_{1}$ is calculated by

(7) $$\alpha_{1}=1.5\cdot exp\left(-{\left(\frac{t}{T_{max}}\right)}^{2}\right)$$

where *t* indicates the current iteration, and $T_{max}$ is the maximum number. In the early stage of the honey badger search, a larger $\alpha_{1}$ value ($\alpha_{1}>1$) can help followers obtain a better search space. In the later stage of the honey badger search, reducing the $\alpha_{1}$ value ($\alpha_{1}<1$) can help followers search for the optimal value of the optimization problem.

Logistic mapping is widely used in swarm intelligence optimization. Its advantage lies in taking advantage of the ergodicity, randomness, and initial value sensitivity of chaos, which can effectively enhance the global search ability of the algorithm. The logistic mapping is defined as

(8) $$Chaos_{i}=\mu \cdot Chaos_{i-1}\cdot \left(1-Chaos_{i-1}\right),$$

where $Chaos_{i}$ denotes the *i*-th input value of the chaotic system, $Chaos_{i-1}$ indicates the *i*-1-th output value, and μ is the chaotic parameter, where μ=4 in this study.

### 3.2. Exploration Enhancements

The modified digging phase (rand<0.5) and honey phase (rand≥0.5) for the position updating are defined as

(9) $$X_{\mathrm{new}}=\left\{\begin{array}{ccc} \begin{array}{c} \mathrm{Chaos}\cdot r_{1}\cdot X_{\mathrm{prey}}+F\times \Phi \times I\times X_{\mathrm{prey}} \end{array} & \mathrm{if}\;\mathrm{rand}<0.5 & \left(9\text{-}1\right)\\ \;\;+F\times \alpha_{1}\times d_{i}\times \left|\cos\left(2\pi \cdot r_{2}\right)\times \left[1-\cos\left(2\pi \cdot r_{3}\right)\right]\right|,\\ \mathrm{Chaos}\cdot X_{\mathrm{prey}}+F\times r_{4}\times \alpha_{1}\times d_{i}, & \mathrm{if}\;\mathrm{rand}\geq 0.5 & \left(9\text{-}2\right) \end{array}\right.$$

where $X_{prey}$ denotes position of the best prey of the badger; $X_{new}$ is the solution of the updating individual; and Φ represents the capability to obtain food (Φ = 6 in this study). *I* denotes the smell intensity of the prey $X_{prey}$. $d_{i}$ measures the distance from the *i*-th honey badger to the prey. $r_{1}$ to $r_{4}$ are different random values in [0, 1]. $\alpha_{1}$ is the update density factor. Variable *F* defines the direction of the search process.

### 3.3. Exploitation Enhancements

For the quantum rotation strategy, each dimension of an agent is named a quantum bit (qubit) in quantum computation, and there are two basic states: |0〉 or |1〉. At any time, the state of a qubit can be considered a linear combination of two basic states, and it is defined as

(10) |ψ〉=α|0〉+β|1〉

where $\alpha^{2}$ and $\beta^{2}$ are, respectively, the probability amplitudes of the “0” and “1” states, and they must satisfy the condition $\alpha^{2}$ + $\beta^{2}$ = 1. Then, a qubit can consequently be expressed as

(11) $$|\psi \rangle =\left[\begin{array}{c} \alpha \\ \beta \end{array}\right]=\left[\begin{array}{c} cos(\theta )\\ sin(\theta ) \end{array}\right],\theta \in \left[0,2\pi \right]$$

To escape local optima and enhance solution quality, the quantum agents’ positions are updated using a quantum rotation gate, defined as

(12) $$\left[\begin{array}{c} cos(\theta_{i,j}^{t+1})\\ sin(\theta_{i,j}^{t+1}) \end{array}\right]=\left[\begin{array}{c} cos\left(\Delta \theta_{i,j}^{t+1}\right)-sin\left(\Delta \theta_{i,j}^{t+1}\right)\\ sin\left(\Delta \theta_{i,j}^{t+1}\right)+cos\left(\Delta \theta_{i,j}^{t+1}\right) \end{array}\right]\cdot \left[\begin{array}{c} cos(\theta_{i,j}^{t})\\ sin(\theta_{i,j}^{t}) \end{array}\right]$$

Then, we define the $cos(\theta_{i,j})$ and $sin(\theta_{i,j})$ in Equation (13) as $cos\left(\theta_{i,j}\right)=\alpha \cdot cos\left(X_{new}\right)-\beta \cdot sin\left(X_{new}\right)$ and $sin\left(\theta_{i,j}\right)=\alpha \cdot sin\left(X_{new}\right)+\beta \cdot cos\left(X_{new}\right)$, where $X_{new}$ is calculated with Equation (9). Thus, the improved CQLHBA updates the positions using the chaotic strategy, Lévy(λ) flight strategy, and quantum rotation strategy, which are calculated by

(13) $$X_{new}=\left\{\begin{array}{cc} & r_{5}\cdot Chaos\cdot RL_{F}\cdot cos\left(\theta_{i,j}^{2}\right)\cdot X_{new}+X_{prey},\;if\;rand>0.5\\ & r_{6}\cdot Chaos\cdot RL_{F}\cdot sin\left(\theta_{i,j}^{2}\right)\cdot X_{new}+X_{prey},\;if\;rand\leq 0.5 \end{array}\right.$$

where $X_{new}$ is the solution of the updating individual, and $X_{prey}$ indicates the position of the prey. The flight strategy $RL_{F}$ is calculated by

(14) $$RL_{F}=0.05\cdot \frac{u\cdot \sigma }{{\left|v\right|}^{1/\lambda }}$$

(15) $$\sigma ={\left(\frac{\Gamma \left(1+\lambda \right)\cdot \sin\left(\frac{\pi \lambda }{2}\right)}{\Gamma \left(\frac{1+\lambda }{2}\right)\cdot \lambda \cdot 2^{\frac{\lambda -1}{2}}}\right)}^{\frac{1}{\lambda }}$$

where *u* and *v* follow a normal distribution, respectively. λ is a parameter of the Lévy(λ) flight, which is set to 1.5 in this study. Γ(·) denotes the gamma function.

### 3.4. Computational Complexity Analysis

The performance and time efficiency of optimization algorithms can be significantly affected by the specific test platforms employed, meaning that a thorough evaluation of the proposed CQLHBA is essential. To quantitatively assess its efficiency, the computational complexity of the algorithm is analyzed below [23]. Let *N* denote the population size, $T_{max}$ represent the maximum number of iterations, and *D* stand for the dimensionality of the problem. The breakdown of the computational complexity is as follows: initializing the honey badger population demands O(ND) operations; updating positions during the global and local search stages incurs a complexity of O(NDlogD); additionally, the hybrid chaotic, quantum rotation and Lévy flight strategies introduce an extra 2O(NDlogD) to the update process. Moreover, the fitness evaluation and sorting step requires O(NlogN) computations. Taking all these components into account, the total computational complexity of the CQLHBA algorithm can be formulated as

(16) $$O\left(CQLHBA\right)=O\left(ND\right)+O\left(T_{max}\right)\cdot \left(O\left(NDlogD\right)+2\cdot O\left(NDlogD\right)+O\left(NlogN\right)\right)$$

When evaluating the efficiency of the standard HBA, it is essential to analyze its computational cost. The complexity of the basic HBA can be characterized as follows:

(17) $$O\left(HBA\right)=O\left(ND\right)+O\left(T_{max}\right)\cdot \left(O\left(NDlogD\right)+O\left(NlogN\right)\right)$$

### 3.5. Flowchart and Pseudo-Code of the Designed CQLHBA

**Figure 1:**
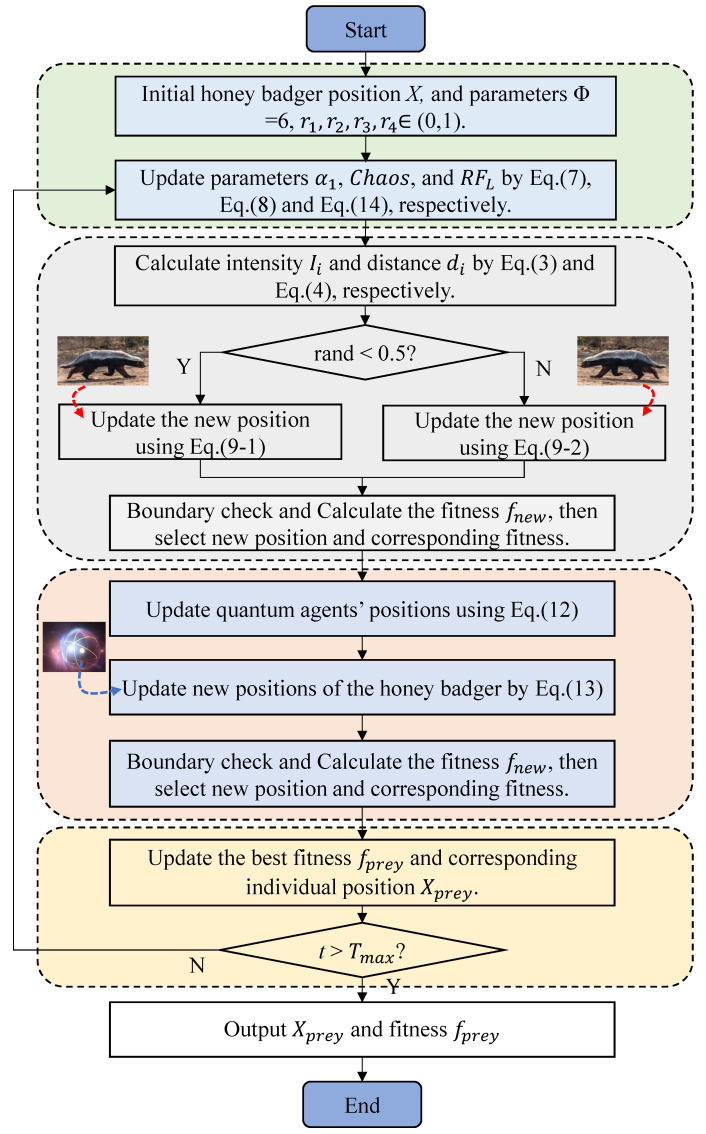
The flowchart of the designed CQLHBA.

Figure 1 depicts the flowchart of the CQLHBA, providing a comprehensive outline of its optimization mechanism. As illustrated in Figure 1, the entire procedure can be broken down into four distinct phases. The four main stages are marked with different colors, respectively. In the initial phase, the positions of the honey badger population are initialized. The second phase encompasses the updating of algorithmic parameters, along with the refinement of the digging and honey positions within the proposed CQLHBA framework. Notably, Equation (9-1) is obtained from Equation (9) with rand<0.5. In addition, Equation (9-2) is obtained from Equation (9) when rand≥0.5. During the third phase, individual positions are further adjusted via quantum rotation and Lévy flight strategies. The final phase corresponds to the identification of the optimal population of honey badgers in terms of fitness over the course of the optimization process. Upon completing the maximum number of iterations ($T_{max}$) of the CQLHBA, both the best solution obtained and its corresponding fitness value are output as the final results.

To provide a comprehensive view of the method’s main architecture, Algorithm 1 presents the pseudo-code of the proposed chaotic quantum-inspired leader honey badger algorithm (CQLHBA). The algorithm is formally delineated, specifying its required input parameters, the expected output (i.e., the best solution $X_{prey}$ found), and the step-by-step core computational procedures that constitute the optimization process.

**Algorithm 1:** Pseudo-code of CQLHBA

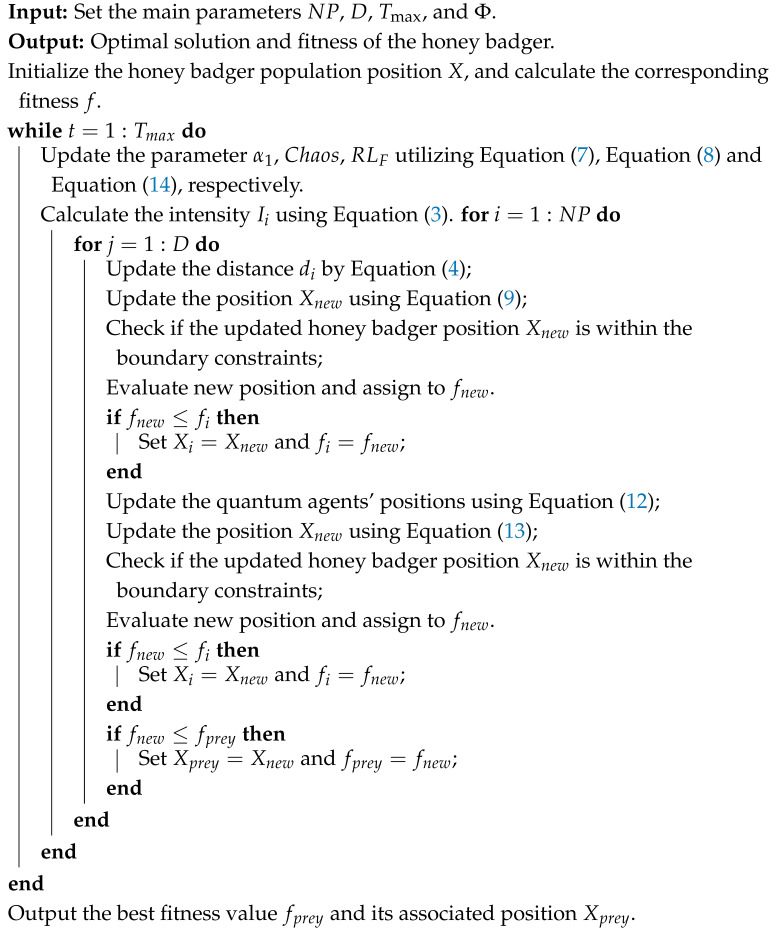



## 4. Results and Analysis

This section provides a comprehensive overview of the experimental framework designed to evaluate the proposed methodology. It elaborates on the configuration employed, which encompasses the suite of benchmark functions selected for testing, the specific hyperparameter settings tuned for the algorithms, and a detailed presentation of the ensuing results. These results are drawn from rigorous evaluations on CEC benchmark suites and are further supplemented by statistical analysis via boxplots.

### 4.1. Benchmark Test Functions

The performance of the proposed algorithm was rigorously evaluated using a comprehensive set of 21 benchmark functions from the CEC test suites [24,25,26]. The evaluation framework comprises six unimodal (F1–F6) and four multimodal (F7–F10) functions from the literature, supplemented with five functions (F11–F15) from CEC 2017 [25] and six functions (F16 to F21) from CEC2022 [26] to ensure diversity. The specific details of the twenty-one functions are itemized in Table 3. To examine scalability, the dimensions for functions F1 to F15 were configured at both 30 and 100. All simulations were conducted in the MATLAB R2018a environment, running on a Windows 10 platform equipped with 16 GB of RAM and an Intel(R) Core(TM) i5-10210U CPU @ 2.11 GHz.

### 4.2. Hyperparameter Settings

An empirical analysis was conducted to quantify the performance of the newly designed CQLHBA. This involved testing the algorithm on a carefully chosen suite of 21 benchmark functions, designed to probe various aspects of its optimization performance, such as solution quality, convergence speed, and scalability. The comparison algorithms were the Honey Badger Algorithm (HBA) [8], Chaotic Sea-Horse Optimizer (CSHO) [27], Sine–Cosine Quantum Salp Swarm Algorithm (SCQSSA) [28], Golden Jackal Optimization (GJO) [29], Aquila Optimizer (AO) [30], Butterfly Optimization Algorithm (BOA) [31], Salp Swarm Algorithm (SSA) [32], Grey Wolf Optimizer (GWO) [6], Randomised Particle Swarm Optimizer (RPSO) [33], and the designed CQLHBA. To maintain consistency across the comparative study, the hyperparameters for each algorithm, as listed in Table 4, were carefully set. A standard population size of NP=30 was employed for all approaches. To ensure the statistical reliability of the results, 30 independent trials were conducted for every test function. The stopping condition for all optimizations was defined by a maximum generation count, set to $T_{max}=1000$ in this work.

### 4.3. Analysis of CEC Benchmark Functions Results

In this subsection, to validate the performance of the proposed algorithm, experimental results in different dimensions (Dim = 30, 100) are presented through comparisons with nine other original and improved algorithms. These include tables of experimental results, convergence curves, and boxplots. Additionally, bar charts are plotted based on the average rankings from the Friedman test. These tests employed a pair of competing hypotheses, the null and the alternative, to evaluate statistical significance. Based on the test results, the null hypothesis was retained when the *p*-value exceeded the significance level; otherwise, the alternative hypothesis was supported.

#### 4.3.1. Ablation Result Analysis

To systematically evaluate the contribution of each proposed strategy, an ablation study was conducted, with the results summarized in Table 5. The specific algorithmic variants are defined as follows: CQLHBA1 denotes the algorithm that incorporates only the quantum rotation strategy. CQLHBA2 corresponds to the version that use solely the quantum rotation strategy and nonlinear dynamic strategy $\alpha_{1}$. CQLHBA3 represents the configuration with three strategies: quantum rotation, Lévy flight, and nonlinear dynamic $\alpha_{1}$. CQLHBA4 represents the configuration with three strategies: quantum rotation, nonlinear dynamic $\alpha_{1}$, and the chaotic dynamic strategy. CQLHBA5 indicates the configuration with two strategies: nonlinear dynamic $\alpha_{1}$ and the chaotic dynamic strategy. For benchmark comparison, the baseline HBA and the fully integrated CQLHBA, which synthesizes all proposed strategies, are also included. As shown in Table 5, all variants (CQLHBA1 to CQLHBA5) demonstrate superior performance compared to the basic HBA, confirming the effectiveness of the individual improvement strategies. Notably, the complete CQLHBA model achieves the best overall performance, indicating that the synergistic integration of multiple strategies can effectively enhance the algorithm’s optimization capability, with each component contributing a complementary effect.

#### 4.3.2. Sensitivity Result Analysis

**Figure 2:**
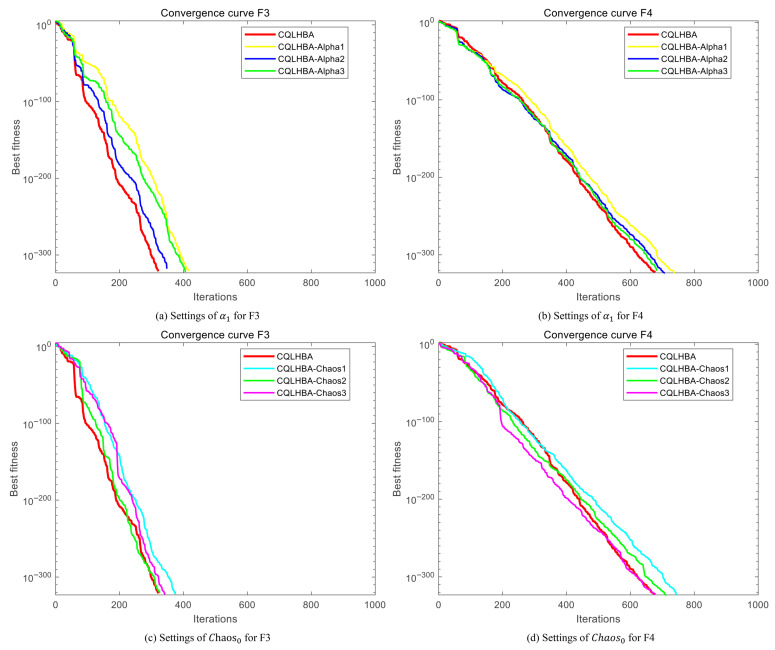
Curves of parameters $\alpha_{1}$ and $Chaos_{0}$ sensitivity analysis for F3 and F4.

To analyze the influence of parameter settings on the numerical optimization results, Table 6 lists the optimization results for *F*3 and *F*4. The settings for the hyperparameter nonlinear dynamic $\alpha_{1}$ are 1.0, 1.8, and 2.0, respectively, corresponding to CQLHBA-Alpha1, CQLHBA-Alpha2, and CQLHBA-Alpha3. For hyperparameter $Chaos_{0}$, we set it to 0.3, 0.4, and 0.45, respectively, corresponding to CQLHBA-Chaos1, CQLHBA-Chaos2, and CQLHBA-Chaos3. As shown in Table 6, all variants (CQLHBA-Alpha1 to CQLHBA-Alpha3, CQLHBA-Chaos1 to CQLHBA-Chaos3) demonstrate superior performance compared to CQLHBA. As can be seen from Table 5 and Table 6, the values of Best, Worst, Mean, and Std are all the same as those of the proposed method. Due to the width of the page, Table 6 lists the results of the parameter sensitivity analysis. The results of the original CQLHBA are detailed in Table 5. In addition, a comparison graph of convergence curves was drawn, as detailed in Figure 2.

From Figure 2, when the constant of the nonlinear dynamic $\alpha_{1}$ strategy is set to 1.5, the number of iterations of CQLHBA is slightly lower than that of CQLHBA-alpha1, CQLHBA-alpha2, and CQLHBA-alpha3 for optimizing F3 and F4 to obtain the optimal value. For the $Chaos_{0}$ of the chaotic dynamic strategy, when $Chaos_{0}$ is set to 0.43, the number of iterations of CQLHBA is slightly lower than that of CQLHBA-Chaos1, CQLHBA-Chaos2, and CQLHBA-Chaos3 for optimizing F3 to obtain the optimal value. However, it has almost the same number of iterations as CQLHBA-Chaos3 for F4, where $Chaos_{0}$ is set to 0.45. Based on the above analysis, CQLHBA parameters $\alpha_{1}$ and $Chaos_{0}$ are set to 1.5 and 0.43.

#### 4.3.3. Result Analysis with Dim = 30

As can be observed from Table 7 with Dim = 30, the proposed CQLHBA achieves the theoretical optimum on functions F1, F2, F3, F4, F7, and F9, with its mean and Std being the best among all ten compared algorithms. Compared to the HBA, the proposed CQLHBA requires a longer optimization time, indicating that the enhancement in algorithm performance comes at the cost of increased computational complexity, which is considered acceptable. For F5, SSA yields the best optimization result among the compared algorithms, with a mean of 1.27 × $10^{-8}$ and an Std of 2.43 × $10^{-9}$. For F6, the SCQSSA achieves the best optimization result, with a mean of 3.43 × $10^{-5}$ and an Std of 3.03 × $10^{-5}$. In addition, CQLHBA, SCQSSA, and AO yield identical results, with a mean of 8.88 × $10^{-16}$ and an Std of 0.00× $10^{0}$ for F8, while SCQSSA has the longest optimization time. For F9, the proposed CQLHBA, along with HBA, CSHO, SCQSSA, GJO, and AO, all achieve the theoretical optimum. For F10, although CQLHBA does not reach the theoretical optimum, its optimization results are superior to all other compared methods, with a mean of 7.41 × $10^{-125}$ and an Std of 4.06 × $10^{-124}$. For F12, F13, and F15, the optimization results of CQLHBA are better than those of HBA. However, for F11 and F14, the optimization results of CQLHBA are slightly inferior to those of HBA. The reason is that the improvement may have interfered with the individual optimization direction of the basic HBA, and thus no better results were obtained. This will be analyzed theoretically in the in-depth study of CQLHBA improvement. It is noteworthy that RPSO achieves better optimization results for F12, F13, and F14 compared to the other methods. According to the Friedman test results in Table 8, the overall ranking of the ten comparison approaches is CQLHBA > HBA > AO > CSHO > GJO > GWO > SCQSSA > RPSO > SSA > BOA.

**Figure 3:**
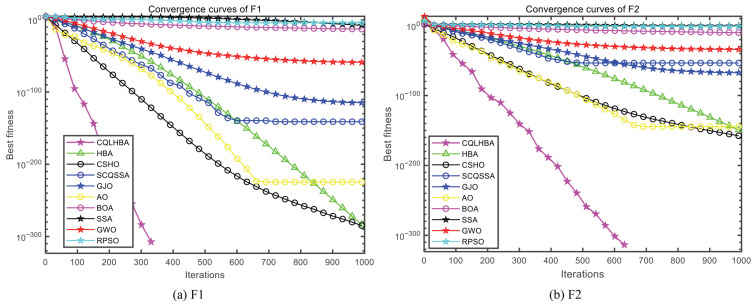

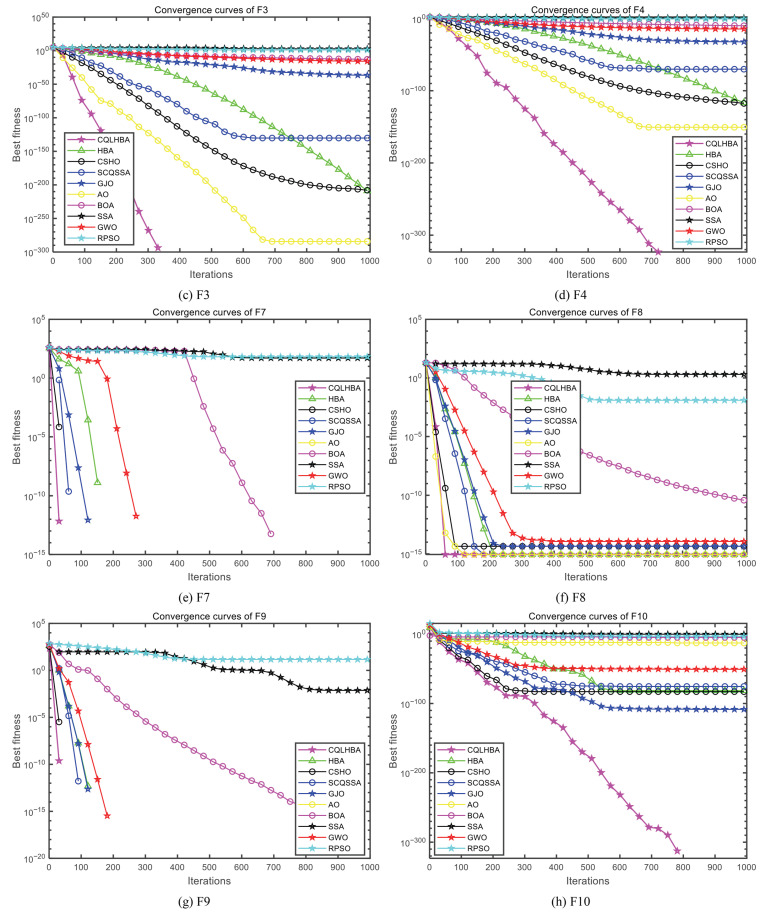
Curves of the ten comparison algorithms with Dim = 30.

Figure 3 illustrates the convergence curves of the compared algorithms with Dim = 30, presenting the results for F1 to F4 and F7 to F10. Except for F7 and F9, the proposed CQLHBA achieves the theoretical optimum in fewer iterations, demonstrating its superior convergence speed and generalization capability. For the convergence curves of F7 and F9, the iteration count of the AO algorithm is comparable to that of CQLHBA, with their curves nearly overlapping.

**Figure 4:**
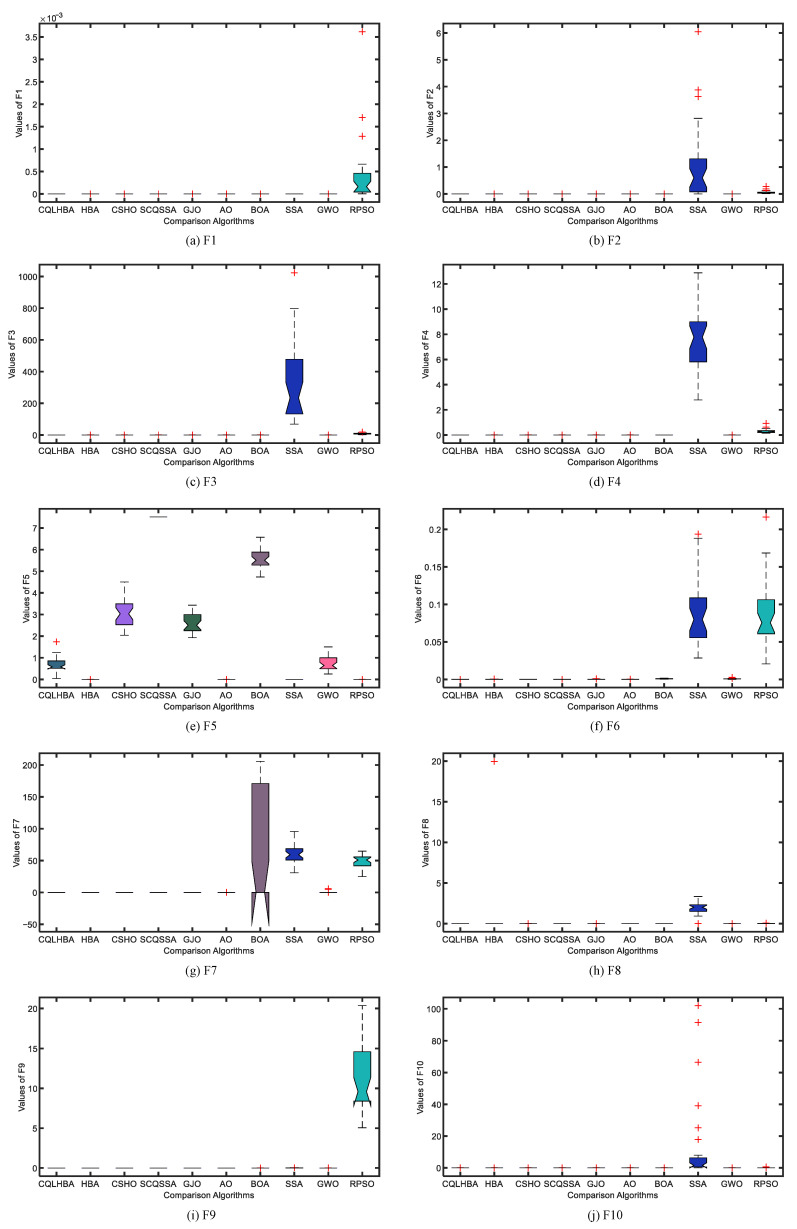
Boxplots of the ten comparison algorithms from F1 to F10 with Dim = 30.

**Figure 5:**
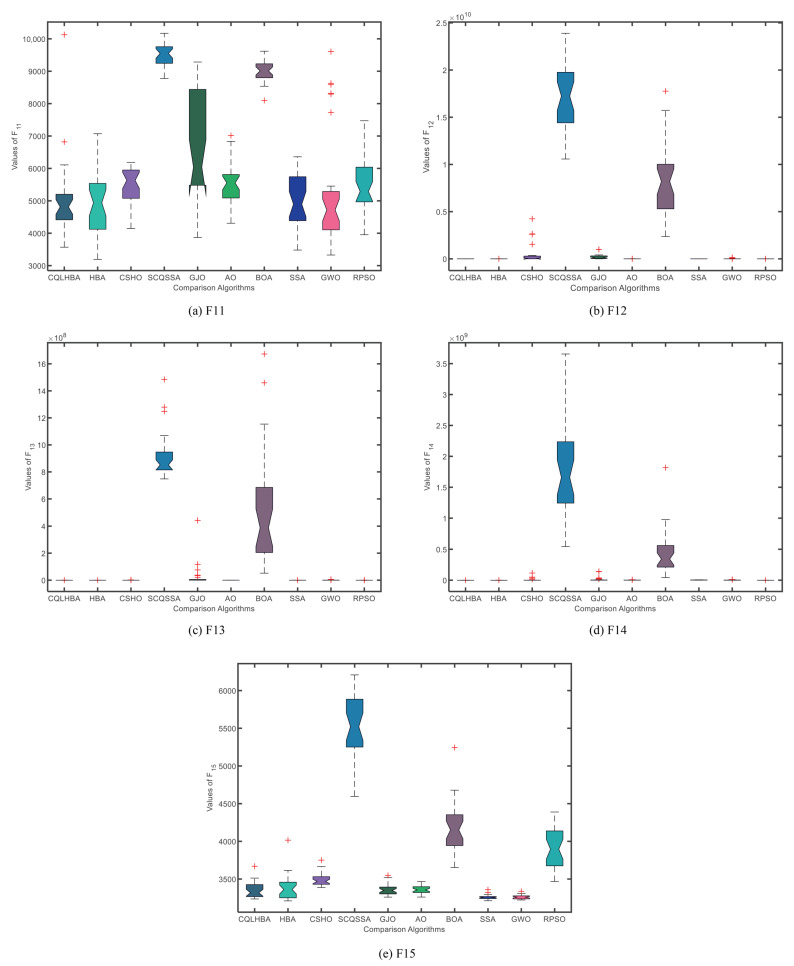
Boxplots of the ten comparison algorithms from F11 to F15 with Dim = 30.

To better illustrate the stability of the compared algorithms, Figure 4 and Figure 5 display the boxplots of the comparative methods. As can be observed from Figure 4, the designed CQLHBA exhibits better stability on F1, F2, F3, and F4 compared to other methods. For F7, F8, and F9, although CQLHBA shows good stability, the other compared methods also demonstrate satisfactory performance in this regard. Regarding F5, the boxplot of SSA indicates the best stability. Based on the data from Table 7 and Figure 4 and Figure 5, it is evident that the optimization performance of the proposed CQLHBA on F5, F11, and F15 requires further improvement.

**Figure 6:**
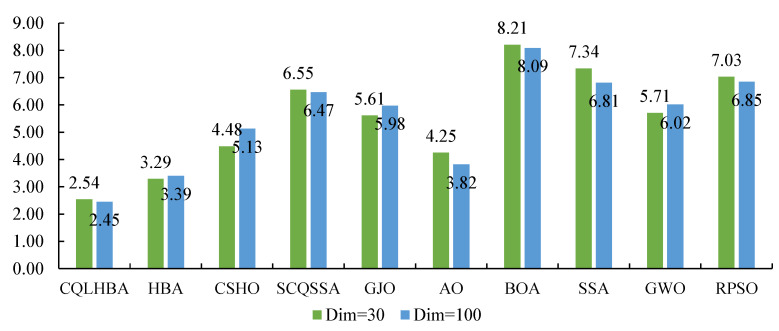
Mean rank of the ten comparison methods by the Friedman test with Dim 30 and 100.

Furthermore, according to Table 8 and Table 9, Figure 6 presents the bar chart of the mean Friedman test results for optimization outcomes across different dimensions. This chart clearly shows the ranking of the compared methods, where lower values indicate better overall algorithm performance.

#### 4.3.4. Result Analysis with Dim = 100

Table 7 and Table 10 provide statistical results, including the mean, standard deviation (Std), training time (Time/s), and *p*-value based on Wilcoxon’s signed-rank (WSR) test [34], while Table 8 and Table 9 list the ranked results of the Friedman test, respectively. The significance level for the WRS test was established at 0.05. As can be observed from Table 10 with Dim = 100, the proposed CQLHBA achieves the theoretical optimum on functions F1, F2, F3, F4, F7, and F9, with its mean and Std being the best among all ten compared algorithms. The superior performance of CQLHBA is achieved at the expense of increased computational complexity relative to HBA, a compromise deemed acceptable. For F5, AO obtains the best optimization result among the compared algorithms, with a mean of 9.34 × $10^{-5}$ and an Std of 1.44 × $10^{-4}$. For F6, the SCQSSA achieves the best optimization result, with a mean of 4.01 × $10^{-5}$ and an Std of 4.38 × $10^{-5}$, and its optimization time is 2.91 × $10^{0}$ s, which is higher than CQLHBA. In addition, CQLHBA, SCQSSA, and AO also yield identical results, with a mean of 8.88 × $10^{-16}$ and an Std of 0.00× $10^{0}$ for F8, while SCQSSA has the longest optimization time. For F9, the proposed CQLHBA reaches the theoretical optimum, a level of accuracy that is also achieved by its peers HBA, CSHO, SCQSSA, GJO, AO, and GWO. This outcome demonstrates that for this specific function, these methods exhibit equivalent and maximal effectiveness in locating the mathematical optimum. For F10, although CQLHBA does not reach the theoretical optimum, its optimization results are superior to all other compared methods, with a mean of 2.94 × $10^{-187}$ and an Std of 0.00× $10^{0}$. In addition, the mean values of BOA, SSA, and RPSO for F10 seriously exceed the threshold of optimization, resulting in the phenomenon of dimensional disaster. For F12, F13, and F15, the optimization results of CQLHBA are also better than those of HBA with Dim = 100. It is notable that the RPSO algorithm secured superior optimization results for functions F12, F13, and F14. This performance stands in contrast to that of the proposed CQLHBA, which was marginally surpassed by the original HBA on functions F11 and F14. From the Friedman test results in Table 9, the overall ranking of the ten comparison approaches is CQLHBA > HBA > AO > CSHO > GJO > GWO > SCQSSA > SSA > RPSO > BOA.

**Figure 7:**
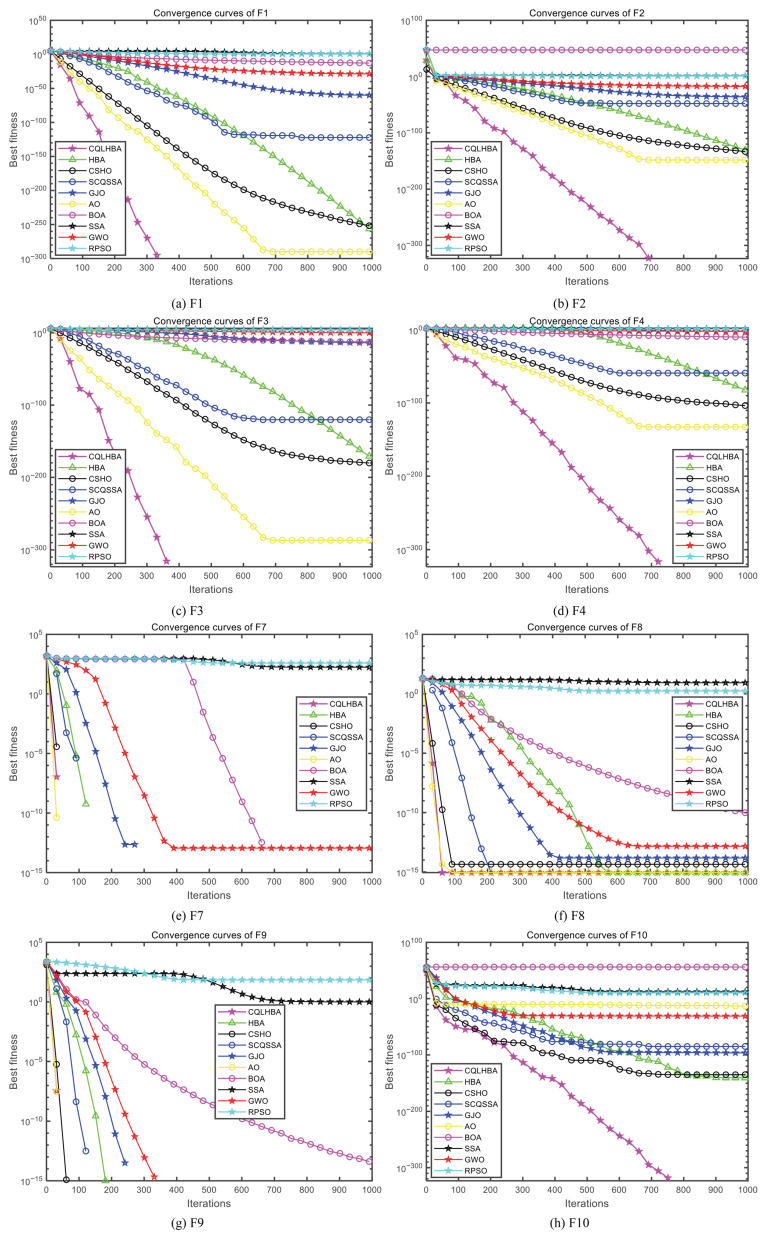
Curves of the ten comparison algorithms with Dim = 100.

**Figure 8:**
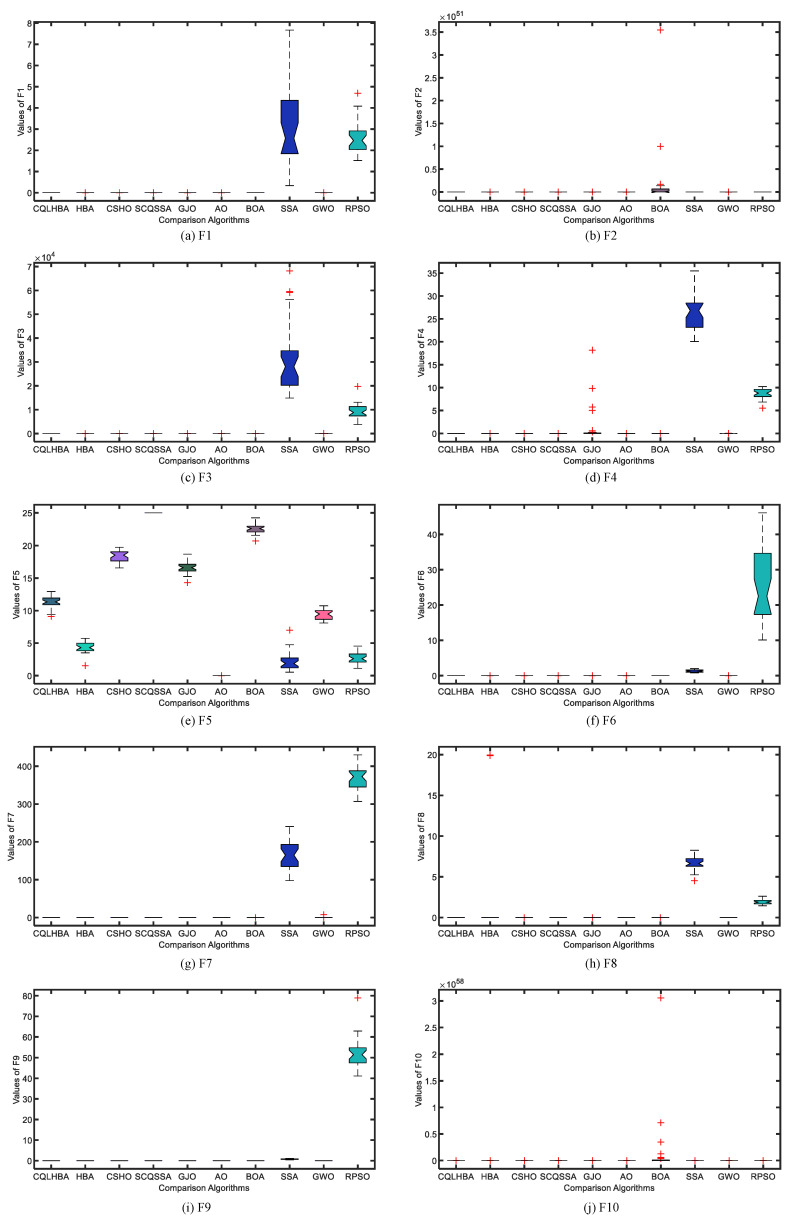
Boxplots of the ten comparison algorithms from F1 to F10 with Dim = 100.

**Figure 9:**
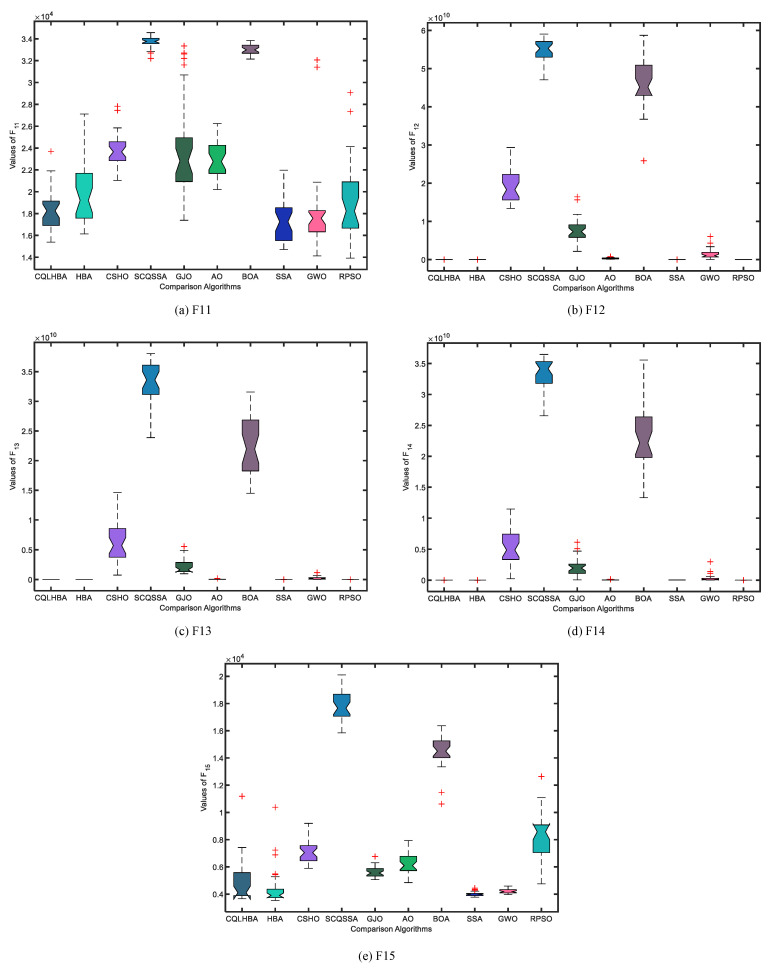
Boxplots of the ten comparison algorithms from F11 to F15 with Dim = 100.

Figure 7 depicts the convergence curves of the compared algorithms with Dim = 100, presenting the results for F1 to F4 and F7 to F10. Except for F8, the proposed CQLHBA can all reach the theoretical optimal value with fewer iterations, indicating that the improved method has better convergence and generalization. For the convergence curves of F7 and F9, the number of iterations of the AO algorithm is slightly lower than that of CQLHBA, and it can reach the theoretical optimal value. Figure 8 and Figure 9 display the boxplots of the ten comparison algorithms. As can be observed from Figure 8, the designed CQLHBA exhibits better stability on F1, F2, F3, and F4 compared to other methods. For F7 to F10, although CQLHBA shows good stability, the other compared methods also demonstrate satisfactory performance in this regard. Regarding F5 with 100 dimensions, the boxplot of the AO algorithm indicates the best stability. Based on the data from Table 10 and Figure 8 and Figure 9, it is evident that the optimization performance of the proposed CQLHBA on F5, F11, and F15 requires further improvement.

#### 4.3.5. Result Analysis of Six Test Functions from CEC2022 with Dim = 20

Table 11 presents the experimental results for six benchmark functions from CEC2022 with maximum test dimensions (Dim = 20), where F16 to F21 correspond to CEC2022’s F1, F2, F6, F7, F9, and F10, respectively. The results include the mean, Std, Time/s, and *p*-value based on the WSR test. As observed in Table 11, the proposed CQLHBA consistently outperforms HBA on functions F16 to F19. For F20, CQLHBA and HBA achieve an identical mean value; however, HBA demonstrates a superior Std compared to CQLHBA. Furthermore, HBA yields better optimization results than CQLHBA for F21. It is noteworthy that RPSO achieves the best optimization results among all compared algorithms for functions F16, F17, F18, and F20. Nevertheless, according to the Friedman test in Table 12, the proposed CQLHBA attains the smallest average ranking value, indicating its superior overall comprehensive performance.

**Figure 10:**
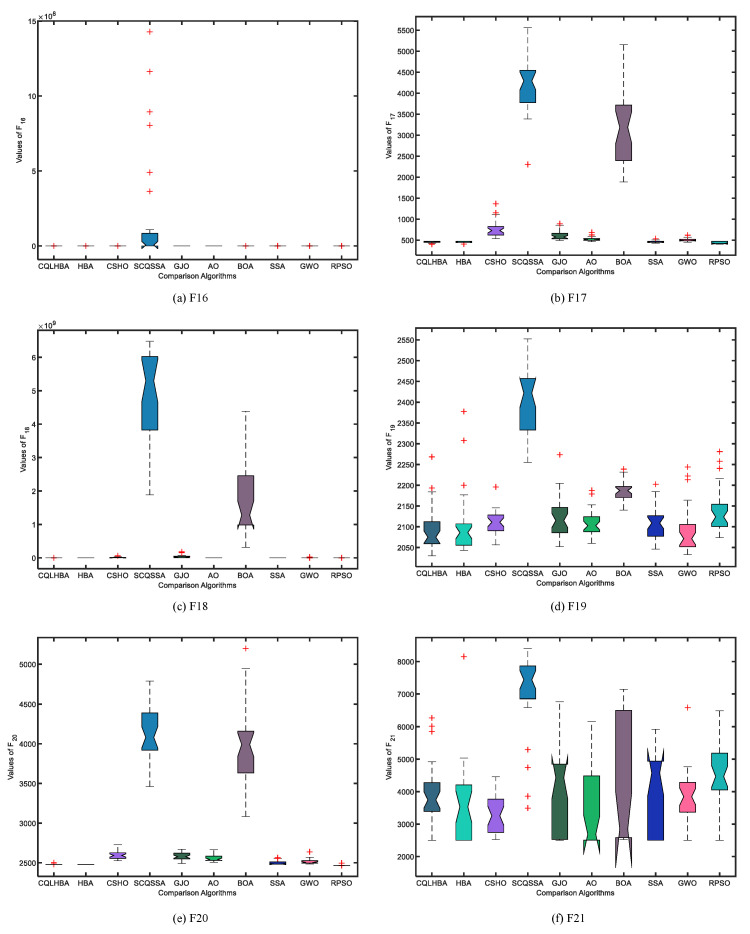
Boxplots of the ten comparison algorithms for the six test functions from CEC2022 with Dim = 20.

**Figure 11:**
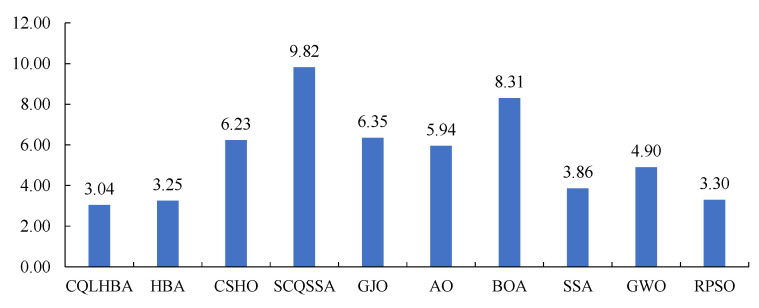
Mean rank of the ten comparison methods by the Friedman test for the six test functions from CEC2022.

Figure 10 shows the boxplots of the ten comparison algorithms for the CEC2022 test functions. From Figure 10, the designed CQLHBA exhibits better stability on F16, F18, F19, and F21 compared to basic HBA. Referring to Table 11 and Figure 10, it is evident that the optimization performance of the proposed CQLHBA on F19 and F21 requires further improvement. In addition, Figure 11 presents the bar chart of the mean Friedman test results of the ten comparison methods. Thus, the overall ranking of the ten comparison approaches is CQLHBA > HBA > RPSO > SSA > GWO > AO > CSHO > GJO > BOA > SCQSSA.

#### 4.3.6. Result Analysis of Engineering Optimization Problems

To verify the effectiveness of the proposed QCLHBA, the three-bar truss design (TBTD) [30,35] and tension/compression spring design (TSD) [6,8] engineering constraint optimization problems were applied in this study. The TBTD problem is a classic optimization problem aimed at minimizing structural forces. Here, design variables $x_{1}$ and $x_{2}$ represent the cross-sectional areas $A_{1}$ and $A_{2}$ of the longer and shorter truss bars, respectively, while *l* denotes the length of each bar. The problem is mathematically formulated as follows:

(18) $$f\left(x_{1},x_{2}\right)=f\left(A_{1},A_{2}\right)=\left(2\sqrt{2A_{1}}+A_{2}\right)\cdot l,$$

subject to

$$g_{1}\left(x\right)=\frac{\sqrt{2}A_{1}+A_{2}}{\sqrt{2}A_{1}^{2}+2A_{1}A_{2}}P-\sigma \leq 0,$$

$$g_{2}\left(x\right)=\frac{A_{2}}{\sqrt{2}A_{1}^{2}+2A_{1}A_{2}}P-\sigma \leq 0,$$

$$g_{3}\left(x\right)=\frac{1}{A_{1}+\sqrt{2}A_{2}}P-\sigma \leq 0.$$

The variable range is

$$0\leq A_{1},\;A_{2}\leq 1,\;l=100\;\mathrm{cm},\;P=2\;{\mathrm{kN}/\mathrm{cm}}^{2},\;\mathrm{and}\;\sigma =2\;{\mathrm{kN}/\mathrm{cm}}^{2}.$$

**Figure 12:**
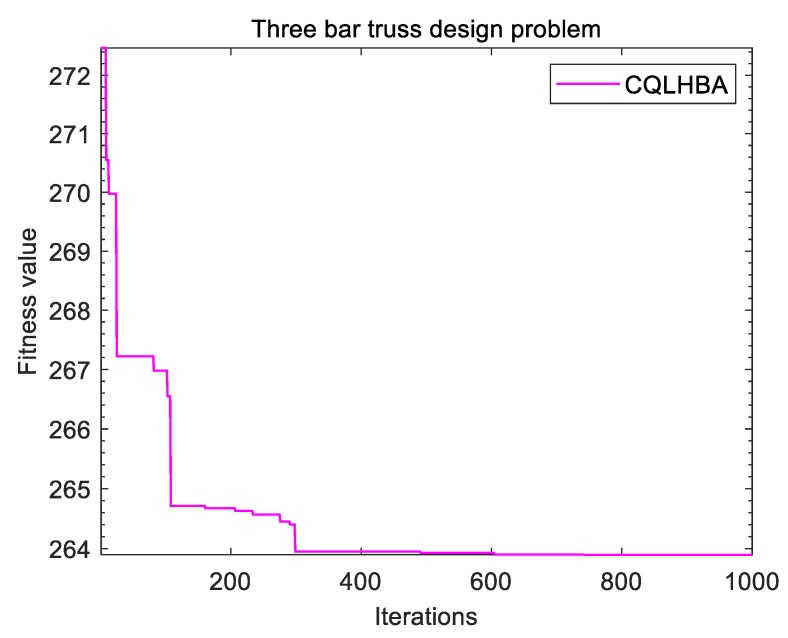
Curve of the CQLHBA for the TBTD problem.

Table 13 lists the optimal value for addressing the TBTD problem with eight comparison algorithms, including PSO, GWO, AO, BOA, GJO, CSHO, HBA, and the designed CQLHBA. CQLHBA obtained the optimal value of 263.8961, corresponding to $x_{1}$ and $x_{2}$ at 0.7887 and 0.4083, respectively. The result of $f_{min}$ retains four decimal places in Table 13. Notably, the HBA also obtained the optimal value of 263.8961 with $x_{1}$ and $x_{2}$ at 0.7881 and 0.4099, respectively. In addition, Figure 12 depicts the fitness curve of the proposed CQLHBA for addressing the TBTD problem.

The TSD [6] is the cost minimization task; $x_{1}$ is the wire diameter, which is *d*; $x_{2}$ denotes the mean coil diameter, which is *D*; and $x_{3}$ is the number of active coils, which is *N*. The objective is to minimize the spring mass, which is governed by the following formulation:

(19) $$f\left(x_{1},x_{2},x_{3}\right)=f\left(d,D,N\right)=x_{1}^{2}\cdot x_{2}x_{3}+2x_{1}^{2}\cdot x_{2},$$

subject to

$$g_{1}\left(x\right)=1-\frac{x_{2}^{3}x_{3}}{71,785x_{1}^{4}}\leq 0,$$

$$g_{2}\left(x\right)=\frac{4x_{1}^{2}-x_{1}x_{2}}{12,566(x_{1}\cdot x_{2}^{3}-x_{2}^{4})}+\frac{1}{5108x_{2}^{2}}-1\leq 0,$$

$$g_{3}\left(x\right)=1-\frac{140.45x_{2}}{x_{1}^{2}x_{3}}\leq 0,$$

$$g_{4}\left(x\right)=\frac{x_{1}+x_{2}}{1.5}-1\leq 0.$$

The variable range is

$$0.25\leq x_{1}\leq 1.3,\;0.05\leq t\leq 2.0,\;\mathrm{and}\;2\leq x_{3}\leq 15.$$

**Figure 13:**
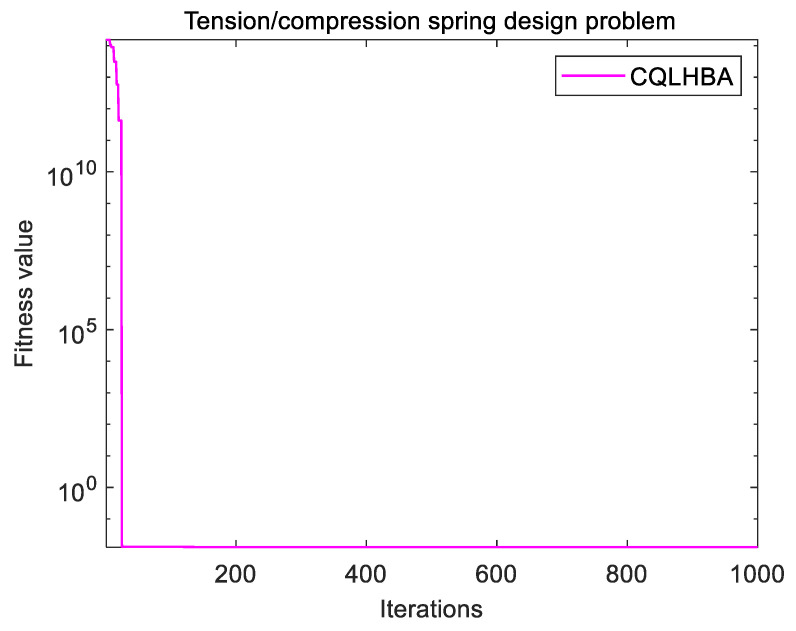
Curve of the CQLHBA for the TSD problem.

Table 14 lists the optimal value for addressing the TSD problem with eight comparison algorithms, including PSO, L-SHADE, WOA, BOA, GJO, CSHO, HBA, and the designed CQLHBA. CQLHBA obtained the optimal value of 0.0127 corresponding to $x_{1}$, $x_{2}$, and $x_{3}$ at 0.0500, 0.3174, and 14.031, respectively. In addition, from $f_{min}$, WOA can obtain the same optimal value of 0.0127 when four decimal places are retained, which corresponds to $x_{1}$, $x_{2}$, and $x_{3}$ at 0.0523, 0.3720, and 10.4470, respectively. In addition, Figure 13 depicts the fitness curve of the proposed CQLHBA for addressing the TSD problem.

## 5. Results of the WSN

### 5.1. Theory of the Node Coverage Optimization (NCO) Problem

The operational domain is postulated to be a two-dimensional plane of side length *L*, within which *n* target detection points are distributed. These points necessitate perception by the sensor nodes, each characterized by two crucial radii: the sensing radius ($R_{s}$) and the communication radius ($R_{c}$), hereafter referred to as $R_{s}$ and $R_{c}$, respectively. A fundamental calculation in this model is the Euclidean distance, denoted as d(i,s), separating a sensor node *i* from a target point *s*, which is given by

(20) $$d\left(i,s\right)=\sqrt{{\left(x_{s}-x_{i}\right)}^{2}+{\left(y_{s}-y_{i}\right)}^{2}}$$

where $\left(x_{i},y_{i}\right)$ and $\left(x_{s},y_{s}\right)$ represent the coordinates of the *i*-th target point and the sensor node *s*, respectively. Following the binary sensing model [20], the coverage probability *p* that a target node *i* is successfully perceived by sensor node *s* is given by

(21) $$p\left(i,s\right)=\{\begin{array}{c} 0,d\left(i,s\right)\geq R_{s},\\ 1,d\left(i,s\right)<R_{s}. \end{array}$$

According to the binary perception model, the two-dimensional deployment area is discretized along the *x*-axis and *y*-axis with a fixed step length, *q*. This partitions the area into a grid of cells, each with a side length of l=q. Consequently, the entire region is divided into $q^{2}$ such grid cells for analysis. The overall coverage rate of the deployed sensor nodes across the work area is then defined as

(22) $$C\mathrm{ov}=\frac{p_{\mathrm{cov}}}{q^{2}}=\frac{\sum_{i=1}^{S}p(i,s)}{q^{2}}.$$

Consequently, the NCO problem, governed by the binary sensing model and its coverage metric, can be abstracted into a constrained optimization framework. This framework is governed by a set of four constraints, formally stated below:

(23) $$\max f\left(x\right)=Cov,s.t\left\{\begin{array}{c} g_{1}=\sum_{i=1}^{S}p(i,s)\geq 0\\ g_{2}=\sum_{i=1}^{S}p\left(i,s\right)-q^{2}\geq 0\\ g_{3}=d\left(i,s\right)-R_{s}\geq 0\\ g_{4}=S-M\geq 0 \end{array}\right.$$

where p(i,s) is the probability that target node *i* is covered by sensor node *s*; $q^{2}$ corresponds to the total number of grid intersections in the discretized deployment space; d(i,s) is the Euclidean distance separating sensor *s* and target *i*; and $R_{s}$ indicates the sensing radius of a node. Furthermore, *M* denotes the theoretical number of nodes required for complete area coverage, calculated under the ideal assumption that the sum of individual sensing areas equals the total work area, while *S* represents the actual number of deployed sensor nodes, with the constraint that S≥M.

### 5.2. Result Analysis of the NCO Problem

For the simulation-based study of the Node Coverage Optimization (NCO) problem, a square deployment area of 100 m × 100 m was defined. The investigations were carried out under two distinct experimental setups:

— Experiment 1: Objective: To analyze the coverage optimization performance of CQLHBA. Setup: 20 nodes were randomly deployed with a sensing radius ($R_{s}$) of 15 m and a communication radius ($R_{c}$) of 30 m. Procedure: The comparison algorithms were run for 100 iterations, and their performance was measured based on execution time and the achieved coverage ratio.
— Experiment 2: Objective: To evaluate CQLHBA’s performance in a denser network configuration. Setup: Forty-five nodes were randomly deployed with $R_{s}=10$ m and $R_{c}=20$ m. Procedure: Similarly, the comparison algorithms were executed for 100 iterations for performance analysis.

The comprehensive pseudo-code implementing the proposed CQLHBA for the NCO issue is presented in Algorithm 2.

**Algorithm 2:** Pseudo-code of CQLHBA for the NCO issue

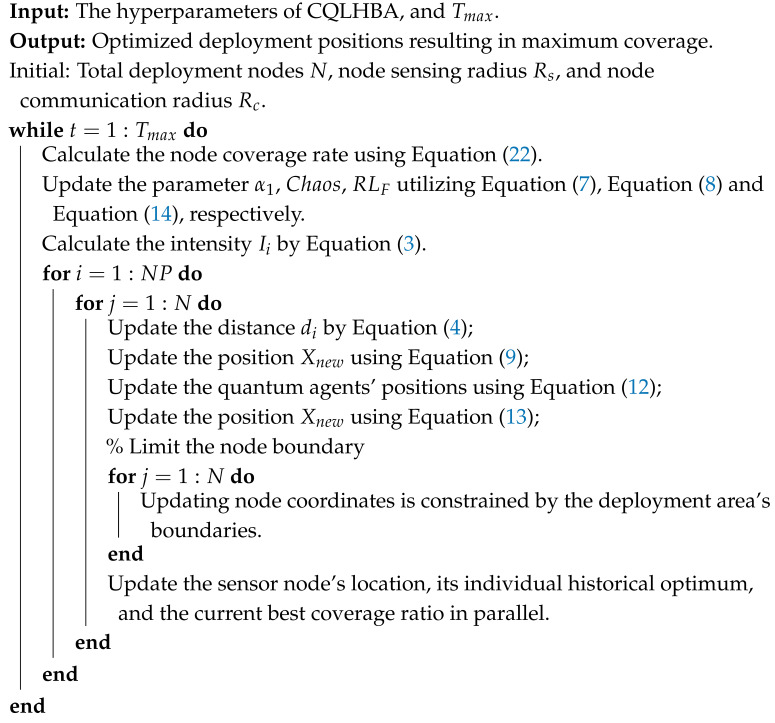



#### 5.2.1. NCO with 15 Nodes in WSNs

In order to rigorously evaluate the effectiveness of the proposed CQLHBA in addressing the Node Coverage Optimization (NCO) problem, a comprehensive comparative analysis was conducted against seven well-established swarm intelligence (SI) algorithms. The selected benchmark algorithms include the original HBA, duck swarm algorithm (DSA) [35], GJO, Marine Predators Algorithm (MPA) [36], GWO, RPSO, and BOA. The experimental configuration for the NCO scenario was defined as follows: a square monitoring region measuring 100 m × 100 m was established, and the performance was tested under two distinct network scales, deploying 20 and 45 sensor nodes, respectively. The maximum number of iterations for all algorithms was consistently set to 100 to ensure a fair computational budget for convergence. Table 15 lists the results of the CQLHBA for the NCO problem.

**Figure 14:**
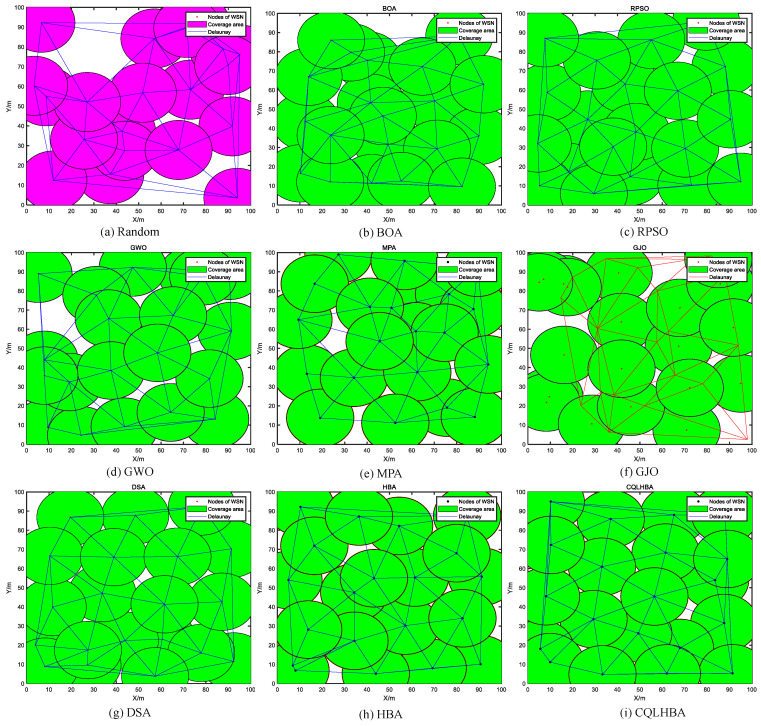
Node coverage rate curves of eight comparison methods with R = 15/m and nodes = 20.

**Figure 15:**
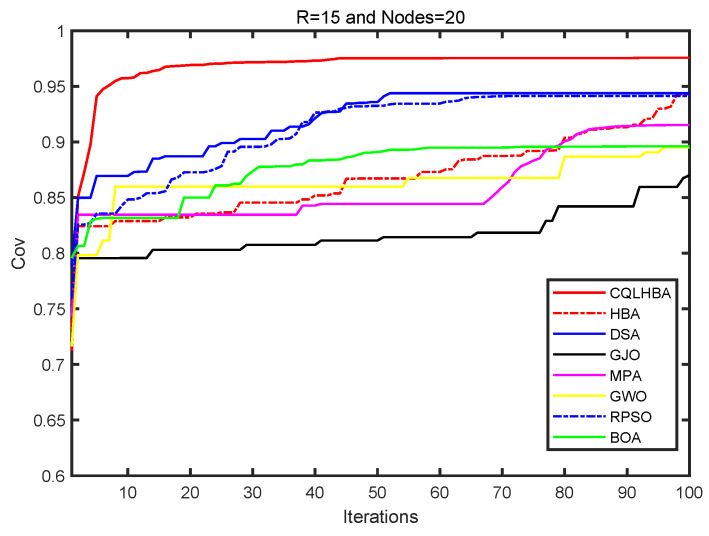
Node coverage rate curves of eight comparison methods with 100 iterations (R = 15, Nodes = 20).

Table 15 shows that for a sensing radius of 15 m with 20 nodes, the results with the proposed CQLHBA are significantly better than with the compared methods. The coverage rate of CQLHBA is 97.57%, the coverage rate for the NCO problem achieved with CQLHBA is higher than that achieved with the other algorithms. The performance of CQLHBA in solving the NCO problems is significantly modified, which indicates that it has high application significance. Compared with the BOA, RPSO, GWO, MPA, GJO, DSA, and HBA, the node coverage rate of CQLHBA with a sensing radius $R_{s}$ of 15 m increased by 7.97 percentage points, 3.44 percentage points, 8.04 percentage points, 6.04 percentage points, 10.55 percentage points, 3.19 percentage points, and 3.29 percentage points, respectively. Figure 14 illustrates the optimized node deployment configurations obtained by the comparative algorithms. Specifically, Figure 14 presents the spatial distribution and corresponding Delaunay triangulation after CQLHBA optimization. The proposed method achieves superior coverage performance with more uniform triangular segmentation, suggesting enhanced communication connectivity and reduced energy consumption among the deployed nodes. Furthermore, Figure 15 displays the coverage convergence curves for all evaluated approaches (BOA, RPSO, GWO, MPA, GJO, DSA, HBA, and CQLHBA).

From Figure 15, the proposed CQLHBA has a significant improvement in coverage in the first 15 iterations. The coverage increase is relatively slow during iterations 15 to 40 and gradually stabilizes after 45 iterations. During the process of coverage improvement, the curve trends of DSA and RPSO are relatively close, and the final coverage of DSA is better than that of RPSO. There is a significant upward trend in the coverage rate of MPA after 70 iterations. The coverage increase of HBA after 50 iterations is greater than before. Of the methods evaluated, GJO, GWO, and BOA demonstrated markedly inferior coverage improvement. The performance of GJO was particularly subpar, resulting in the least significant overall gains.

#### 5.2.2. NCO with 45 Nodes in WSNs

**Figure 16:**
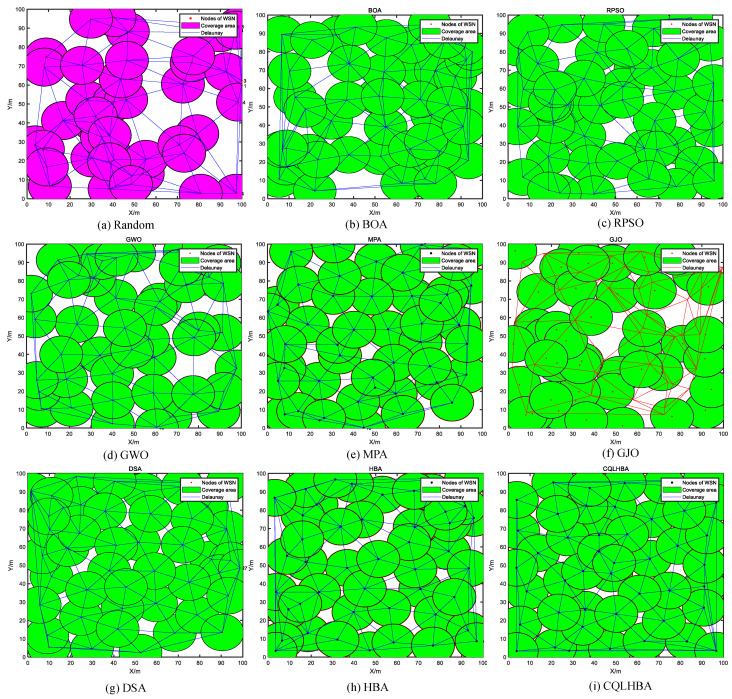
Node coverage rate curves of eight comparison methods with R = 10/m and nodes = 45.

**Figure 17:**
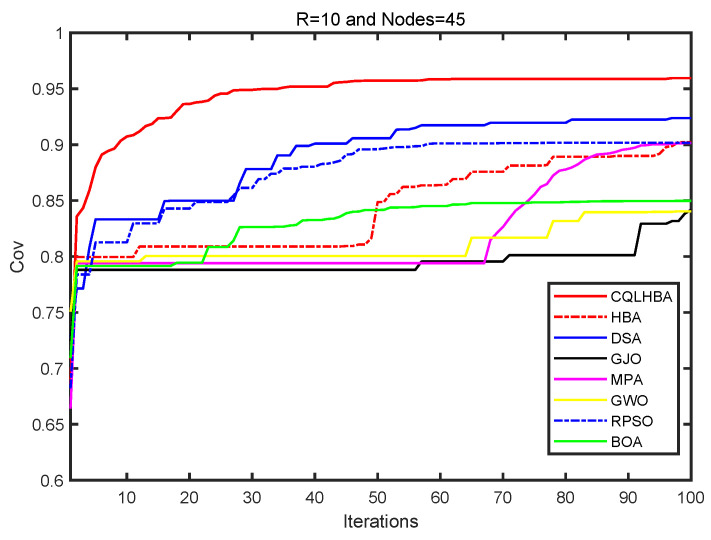
Node coverage rate curves of eight comparison methods with 100 iterations (R = 10, Nodes = 45).

From Table 15, the coverage rate of CQLHBA is 95.94% for a sensing radius of 10 m with 45 nodes, and the coverage rate for the NCO problem achieved with CQLHBA is higher than that achieved with the other algorithms. The performance of CQLHBA in solving the NCO problems is significantly modified, which indicates that it has high application significance. Compared with the BOA, RPSO, GWO, MPA, GJO, DSA, and HBA, the node coverage rate of CQLHBA with a sensing radius $R_{s}$ of 10 m increased by 10.97 percentage points, 5.78 percentage points, 11.90 percentage points, 5.81 percentage points, 11.76 percentage points, 3.57 percentage points, and 5.71 percentage points, respectively. Figure 16 depicts the optimized node coverage results of the comparison algorithms; Figure 16 shows the deployment result of the CQLHBA with higher coverage and more uniform triangulation. The evolutionary coverage rates achieved by each algorithm (BOA, RPSO, GWO, MPA, GJO, DSA, HBA, and CQLHBA) are further compared in Figure 17.

From Figure 17, the proposed CQLHBA has a significant improvement in coverage in the first 30 iterations. The coverage increase is relatively slow during the 30 to 50 iterations and gradually stabilizes after 50 iterations. The optimization trajectories of DSA and RPSO are closely aligned throughout the coverage enhancement phase, yet the final coverage attained by DSA exceeds that of RPSO. Conversely, MPA’s coverage rate exhibits a notably steeper upward trajectory subsequent to the 70th iteration. At 50 iterations, HBA saw a significant improvement in coverage, but after 80 iterations, the increase in coverage was relatively small. However, the coverage improvement of GJO, GWO, and BOA is relatively small compared to other methods, especially GJO. For the NCO problem, the above-mentioned methods can be studied in depth and effective improvement strategies can be proposed.

## 6. Conclusions

This study introduces a novel chaotic quantum-inspired leader honey badger algorithm designed to augment the optimization capabilities of the original HBA for solving complex NCO problem. The proposed algorithm incorporates several key innovations: an adaptive adjustment strategy for the parameter $\alpha_{1}$ to balance the follower position update process, complemented by the integration of chaotic dynamics, quantum rotation, and Lévy flight strategies. These mechanisms collectively enhance the algorithm’s global exploration prowess, local exploitation precision, and overall stability.

The performance of CQLHBA was rigorously evaluated against a suite of twenty-one benchmark functions and compared with several advanced SI algorithms, including HBA, CSHO, SCQSSA, GJO, AO, BOA, SSA, GWO, and RPSO. Experimental results confirm that CQLHBA achieves superior performance, demonstrating a significant advantage in convergence accuracy and robustness. However, the computational complexity of the proposed CQLHBA needs to be reduced, and its performance in solving high-complexity problems should be enhanced. Besides, two engineering constraint optimization problems were used to verify the effectiveness of the proposed QCLHBA. It mainly employs the chaotic nonlinear strategy. Furthermore, the practical value of CQLHBA was validated through its application to the NCO problem in wireless sensor networks (WSNs) by two different deployment scenarios with 100 m × 100 m, where it delivered excellent results in both coverage rate and network connectivity.

In future work, the proposed CQLHBA also requires enhancement for high-dimensional problems, warranting further research into more effective improvement strategies. Its improved variant should be compared with the advanced differential evolution algorithms, such as L-SHADE and IMODE [37,38,39,40], hybrid GWO-SCA [41], etc. These advanced algorithms have broad application potential in engineering optimization [42], the Internet of Things, and feature selection [43]. Specifically within WSN, SI algorithms are pivotal for solving complex challenges like node deployment, routing, and dynamic networking. A significant and growing research focus is their application to multi-objective NCO, dynamic WSNs, energy harvesting, and 3D WSN scenarios [44], which are critical for demanding environments such as underwater monitoring, mountainous terrain surveillance, and forest fire detection. Advancing SI techniques for these complex three-dimensional spaces remain a key research frontier.

## Figures and Tables

**Table 1:** Summary of proposed HBA variants.

Algorithm	Strategy	Year	Ability
HHBADE [12]	Hybrid differential evolution strategy	2022	Balance global exploratory behavior and local refinement capabilities.
MSHBA [13]	Cubic mapping for initialization, along with random search, elite tangent search, and differential mutation strategies.	2023	Enhance search efficiency and robustness.
LHBA [14]	Lévy flight strategy	2024	Escape local optima and improve convergence accuracy.
CHBA [11]	Tent mapping strategy	2025	Enrich initial population diversity.
DMPHBA [16]	Symbiotic mechanism-based strategy	2025	Preserve diversity throughout the evolutionary process.

**Table 2:** Summary of various SI algorithms for NCO problem of WSN.

Algorithm	Problem	Year	Shortcoming
WPA [19]	NCO	2021	a. Ignore the impact of iterations and sensing radii for coverage; b. Low coverage rate.
HPSBOA [20]	NCO	2022	a. Examine only the impact of sensor nodes for coverage.
PSOMBO [22]	NCO	2024	a. Simple experimental setups; b. Low coverage rate.
ALGWO [21]	NCO	2024	a. Examine only the impact of sensor nodes for coverage; b. Low coverage rate.
HBBWOA [16]	NCO	2025	a. Examine only the impact of sensor nodes for coverage; b. Low coverage rate.

**Table 3:** Twenty-one test functions for the performance evaluation of the comparison algorithms.

Formula	Range	Dim	$f_{\min}$	Category
F1 = $\sum_{i=1}^{Dim}x_{i}^{2}$	[−100,100]	30/100	0	U
F2 = $\sum_{i=1}^{Dim}|x_{i}|+\prod_{i=1}^{Dim}\left|x_{i}\right|$	[−10,10]	30/100	0	U
F3 = $\sum_{i=1}^{Dim}{\left(\sum_{j=1}^{i}x_{j}\right)}^{2}$	[−100,100]	30/100	0	U
F4 = $\max\left\{|x_{i}|,1\leq i\leq Dim\right\}$	[−100,100]	30/100	0	U
F5 = $\sum_{i=1}^{Dim}{\left(x_{i}+0.5\right)}^{2}$	[−100,100]	30/100	0	U
F6 = $\sum_{i=1}^{Dim}ix_{i}^{4}+\mathrm{rand}\left(0,1\right)$	[−1.28,1.28]	30/100	0	U
F7 = $\sum_{i=1}^{Dim}\left(x_{i}^{2}-10\cos\left(2\pi x_{i}\right)+10\right)$	[−5.12,5.12]	30/100	0	M
$\begin{array}{l} F8=-20\exp\left(-0.2\sqrt{\frac{1}{Dim}\sum_{i=1}^{Dim}x_{i}^{2}}\right)-\\ \exp\left(\frac{1}{Dim}\sum_{i=1}^{Dim}\cos\left(2\pi x_{i}\right)\right)+20+e \end{array}$	[−32,32]	30/100	0	M
F9 = $\frac{1}{4000}\sum_{i=1}^{Dim}x_{i}^{2}-\prod_{i=1}^{Dim}\cos\left(\frac{x_{i}}{\sqrt{i}}\right)+1$	[−600,600]	30/100	0	M
F10 = $\sum_{i=1}^{Dim}\left(\mathrm{rand}\left(0,1\right)|x_{i}{|}^{i}\right)$	[−5,5]	30/100	0	M
F11 = Shifted and Rotated Schwefel’s Function	[−100,100]	30/100	1000	M
F12 = Hybrid Functions 3 (N=3)	[−100,100]	30/100	1300	M
F13 = Hybrid Functions 5 (N=4)	[−100,100]	30/100	1500	M
F14 = Hybrid Functions 6 (N=5)	[−100,100]	30/100	1900	M
F15 = Composition Functions 7 (N=6)	[−100,100]	30/100	2700	M
F16 = Shifted and Fully Rotated Zakharov Function	[−100,100]	20	300	U
F17 = Shifted and Fully Rotated Rosenbrock Function	[−100,100]	20	400	M
F18 = Hybrid Function 1 (N=3)	[−100,100]	20	1800	M
F19 = Hybrid Function 2 (N=6)	[−100,100]	20	2000	M
F20 = Composition Function 1 (N=5)	[−100,100]	20	2300	M
F21 = Composition Function 2 (N=4)	[−100,100]	20	2400	M

**Table 4:** Comparison algorithms’ hyperparameter settings.

Algorithms	Hyperparameter
RPSO [33]	$c_{p,max}=c_{g,max}=2.5,c_{p,min}=c_{g,min}=0.5,\omega_{min}=0.2,\omega_{max}=0.9,V_{max}=1.5$
GWO [6]	$a_{first}=2,a_{final}=0$
SSA [32]	$c_{2},c_{3}\in \left(0,1\right)$
BOA [31]	$a=0.1,c\left(0\right)=0.01,p=0.5$
AO [30]	$\alpha =0.1,\delta =0.1,u=0.0265,r_{0}=10$
GJO [29]	$c_{1}=1.5,r_{1}\in \left(0,1\right)$
SCQSSA [28]	$a=2,r=0.5,c_{2},c_{3},c_{4}\in \left(0,1\right)$
CSHO [27]	a=4,u=0.05,v=0.05,β=1.5
HBA [8]	$C=2,\beta =6,r_{1}tor_{4}\in \left(0,1\right)$
CQLHBA	$\Phi =6,\mu =4,Chaos_{0}=0.43,r_{1}tor_{6}\in \left(0,1\right)$

**Table 5:** Ablation results of the proposed strategies of the CQLHBA.

Function	Item	HBA	CQLHBA1	CQLHBA2	CQLHBA3	CQLHBA4	CQLHBA5	CQLHBA
F3	Best	1.15 × $10^{-218}$	7.39 × $10^{-219}$	4.95 × $10^{-254}$	2.04 × $10^{-246}$	0.00 × $10^{0}$	0.00 × $10^{0}$	0.00 × $10^{0}$
	Worst	1.86 × $10^{-202}$	2.32 × $10^{-200}$	1.44 × $10^{-230}$	3.40 × $10^{-232}$	0.00 × $10^{0}$	0.00 × $10^{0}$	0.00 × $10^{0}$
	Mean	7.74 × $10^{-202}$	4.80 × $10^{-232}$	1.14 × $10^{-233}$	0.00 × $10^{0}$	0.00 × $10^{0}$	1.18 × $10^{-203}$	0.00 × $10^{0}$
	Std	2.39 × $10^{-210}$	2.05 × $10^{-211}$	8.57 × $10^{-243}$	6.99 × $10^{-241}$	0.00 × $10^{0}$	0.00 × $10^{0}$	0.00 × $10^{0}$
	Time/s	5.18 × $10^{-1}$	8.88 × $10^{-1}$	8.79 × $10^{-1}$	1.17 × $10^{0}$	9.68 × $10^{-1}$	9.63 × $10^{-1}$	1.12 × $10^{0}$
F4	Best	4.74 × $10^{-125}$	3.08 × $10^{-124}$	2.14 × $10^{-142}$	7.35 × $10^{-141}$	0.00 × $10^{0}$	0.00 × $10^{0}$	0.00 × $10^{0}$
	Worst	1.89 × $10^{-117}$	1.41 × $10^{-116}$	5.84 × $10^{-134}$	1.28 × $10^{-133}$	0.00 × $10^{0}$	0.00 × $10^{0}$	0.00 × $10^{0}$
	Mean	4.53 × $10^{-118}$	2.63 × $10^{-117}$	1.39 × $10^{-134}$	2.45 × $10^{-134}$	0.00 × $10^{0}$	0.00 × $10^{0}$	0.00 × $10^{0}$
	Std	2.14 × $10^{-120}$	3.96 × $10^{-121}$	1.54 × $10^{-137}$	3.25 × $10^{-137}$	0.00 × $10^{0}$	0.00 × $10^{0}$	0.00 × $10^{0}$
	Time/s	2.59 × $10^{-1}$	0.39406542	4.15 × $10^{-1}$	8.62 × $10^{-1}$	4.63 × $10^{-1}$	4.95 × $10^{-1}$	6.09 × $10^{-1}$

**Table 6:** Sensitivity results of parameters $\alpha_{1}$ and $Chaos_{0}$ of the CQLHBA.

Function	Item	CQLHBA-Alpha1	CQLHBA-Alpha2	CQLHBA-Alpha3	CQLHBA-Chaos1	CQLHBA-Chaos2	CQLHBA-Chaos3
F3	Best	0.00 × $10^{0}$	0.00 × $10^{0}$	0.00 × $10^{0}$	0.00 × $10^{0}$	0.00 × $10^{0}$	0.00 × $10^{0}$
	Worst	0.00 × $10^{0}$	0.00 × $10^{0}$	0.00 × $10^{0}$	0.00 × $10^{0}$	0.00 × $10^{0}$	0.00 × $10^{0}$
	Mean	0.00 × $10^{0}$	0.00 × $10^{0}$	0.00 × $10^{0}$	0.00 × $10^{0}$	0.00 × $10^{0}$	0.00 × $10^{0}$
	Std	0.00 × $10^{0}$	0.00 × $10^{0}$	0.00 × $10^{0}$	0.00 × $10^{0}$	0.00 × $10^{0}$	0.00 × $10^{0}$
	Time	9.04 × $10^{-1}$	9.72 × $10^{-1}$	9.53 × $10^{-1}$	9.80 × $10^{-1}$	9.46 × $10^{-1}$	9.87 × $10^{-1}$
F4	Best	0.00 × $10^{0}$	0.00 × $10^{0}$	0.00 × $10^{0}$	0.00 × $10^{0}$	0.00 × $10^{0}$	0.00 × $10^{0}$
	Worst	0.00 × $10^{0}$	0.00 × $10^{0}$	0.00 × $10^{0}$	0.00 × $10^{0}$	0.00 × $10^{0}$	0.00 × $10^{0}$
	Mean	0.00 × $10^{0}$	0.00 × $10^{0}$	0.00 × $10^{0}$	0.00 × $10^{0}$	0.00 × $10^{0}$	0.00 × $10^{0}$
	Std	0.00 × $10^{0}$	0.00 × $10^{0}$	0.00 × $10^{0}$	0.00 × $10^{0}$	0.00 × $10^{0}$	0.00 × $10^{0}$
	Time	5.62 × $10^{-1}$	5.24 × $10^{-1}$	5.34 × $10^{-1}$	5.23 × $10^{-1}$	5.32 × $10^{-1}$	6.71 × $10^{-1}$

**Table 7:** Results of ten algorithms using 15 benchmark functions with Dim = 30.

Function	Item	CQLHBA	HBA	CSHO	SCQSSA	GJO	AO	BOA	SSA	GWO	RPSO
F1	Mean	0.00× $10^{0}$	7.53 × $10^{-276}$	4.49 × $10^{-286}$	7.16 × $10^{-130}$	1.12 × $10^{-111}$	3.36 × $10^{-197}$	1.18 × $10^{-13}$	1.28 × $10^{-8}$	5.63 × $10^{-59}$	4.03 × $10^{-4}$
	Std	0.00× $10^{0}$	0.00× $10^{0}$	0.00× $10^{0}$	2.16 × $10^{-129}$	5.59 × $10^{-111}$	0.00× $10^{0}$	8.60 × $10^{-15}$	3.23 × $10^{-9}$	1.08 × $10^{-58}$	7.18 × $10^{-4}$
	Time/s	6.04 × $10^{-1}$	2.43 × $10^{-1}$	4.43 × $10^{-1}$	8.26 × $10^{-1}$	2.59 × $10^{-1}$	2.54 × $10^{-1}$	1.23 × $10^{-1}$	1.56 × $10^{-1}$	1.84 × $10^{-1}$	7.49 × $10^{-2}$
	*p*-value	/	1.73 × $10^{-6}$	1.73 × $10^{-6}$	1.73 × $10^{-6}$	1.73 × $10^{-6}$	1.73 × $10^{-6}$	1.73 × $10^{-6}$	1.73 × $10^{-6}$	1.73 × $10^{-6}$	1.73 × $10^{-6}$
F2	Mean	0.00× $10^{0}$	4.35 × $10^{-146}$	1.09 × $10^{-158}$	3.66 × $10^{-52}$	3.84 × $10^{-66}$	1.20 × $10^{-105}$	6.16 × $10^{-11}$	1.05 × $10^{0}$	1.17 × $10^{-34}$	6.18 × $10^{-2}$
	Std	0.00× $10^{0}$	1.24 × $10^{-145}$	2.59 × $10^{-158}$	9.25 × $10^{-52}$	8.70 × $10^{-66}$	6.58 × $10^{-105}$	1.04 × $10^{-11}$	1.41 × $10^{0}$	1.12 × $10^{-34}$	5.64 × $10^{-2}$
	Time/s	6.15 × $10^{-1}$	2.47 × $10^{-1}$	4.57 × $10^{-1}$	8.39 × $10^{-1}$	2.67 × $10^{-1}$	2.62 × $10^{-1}$	1.33 × $10^{-1}$	1.63 × $10^{-1}$	1.93 × $10^{-1}$	7.91 × $10^{-2}$
	*p*-value	/	1.73 × $10^{-6}$	1.73 × $10^{-6}$	1.73 × $10^{-6}$	1.73 × $10^{-6}$	1.73 × $10^{-6}$	1.73 × $10^{-6}$	1.73 × $10^{-6}$	1.73 × $10^{-6}$	1.73 × $10^{-6}$
F3	Mean	0.00× $10^{0}$	7.74 × $10^{-202}$	7.18 × $10^{-204}$	9.72 × $10^{-131}$	1.20 × $10^{-35}$	1.34 × $10^{-197}$	9.87 × $10^{-14}$	3.18 × $10^{2}$	3.31 × $10^{-14}$	8.93 × $10^{0}$
	Std	0.00× $10^{0}$	0.00× $10^{0}$	2.39 × $10^{-210}$	4.95 × $10^{-130}$	6.59 × $10^{-35}$	0.00× $10^{0}$	9.17 × $10^{-15}$	2.42 × $10^{2}$	1.56 × $10^{-13}$	4.19 × $10^{0}$
	Time/s	1.12 × $10^{0}$	5.18 × $10^{-1}$	8.29 × $10^{-1}$	1.07 × $10^{0}$	5.33 × $10^{-1}$	7.38 × $10^{-1}$	6.00 × $10^{-1}$	3.95 × $10^{-1}$	4.21 × $10^{-1}$	3.18 × $10^{-1}$
	*p*-value	/	1.73 × $10^{-6}$	1.73 × $10^{-6}$	1.73 × $10^{-6}$	1.73 × $10^{-6}$	1.73 × $10^{-6}$	1.73 × $10^{-6}$	1.73 × $10^{-6}$	1.73 × $10^{-6}$	1.73 × $10^{-6}$
F4	Mean	0.00× $10^{0}$	4.53 × $10^{-118}$	3.41 × $10^{-116}$	2.78 × $10^{-65}$	2.18 × $10^{-33}$	5.12 × $10^{-103}$	7.67 × $10^{-11}$	7.72 × $10^{0}$	1.90 × $10^{-14}$	3.07 × $10^{-1}$
	Std	0.00× $10^{0}$	2.14 × $10^{-120}$	6.58 × $10^{-116}$	8.66 × $10^{-65}$	3.66 × $10^{-33}$	2.80 × $10^{-102}$	4.72 × $10^{-12}$	2.26 × $10^{0}$	2.99 × $10^{-14}$	1.59 × $10^{-1}$
	Time/s	6.09 × $10^{-1}$	2.59 × $10^{-1}$	4.60 × $10^{-1}$	8.31 × $10^{-1}$	2.56 × $10^{-1}$	2.54 × $10^{-1}$	1.23 × $10^{-1}$	1.57 × $10^{-1}$	1.82 × $10^{-1}$	7.38 × $10^{-2}$
	*p*-value	/	1.73 × $10^{-6}$	1.73 × $10^{-6}$	1.73 × $10^{-6}$	1.73 × $10^{-6}$	1.73 × $10^{-6}$	1.73 × $10^{-6}$	1.73 × $10^{-6}$	1.73 × $10^{-6}$	1.73 × $10^{-6}$
F5	Mean	6.88 × $10^{-1}$	1.77 × $10^{-7}$	3.08 × $10^{0}$	7.50 × $10^{0}$	2.59 × $10^{0}$	2.74 × $10^{-5}$	5.58 × $10^{0}$	1.27 × $10^{-8}$	7.31 × $10^{-1}$	2.84 × $10^{-4}$
	Std	3.39 × $10^{-1}$	4.11 × $10^{-7}$	6.59 × $10^{-1}$	0.00× $10^{0}$	4.33 × $10^{-1}$	4.78 × $10^{-5}$	4.56 × $10^{-1}$	2.43 × $10^{-9}$	3.57 × $10^{-1}$	4.74 × $10^{-4}$
	Time/s	5.94 × $10^{-1}$	2.38 × $10^{-1}$	4.50 × $10^{-1}$	8.25 × $10^{-1}$	2.64 × $10^{-1}$	2.53 × $10^{-1}$	1.21 × $10^{-1}$	1.57 × $10^{-1}$	1.82 × $10^{-1}$	7.44 × $10^{-2}$
	*p*-value	/	1.73 × $10^{-6}$	1.73 × $10^{-6}$	1.73 × $10^{-6}$	1.73 × $10^{-6}$	1.73 × $10^{-6}$	1.73 × $10^{-6}$	1.73 × $10^{-6}$	9.59 × $10^{-1}$	1.73 × $10^{-6}$
F6	Mean	3.89 × $10^{-5}$	1.93 × $10^{-4}$	5.79 × $10^{-5}$	3.43 × $10^{-5}$	2.40 × $10^{-4}$	6.63 × $10^{-5}$	8.69 × $10^{-4}$	8.93 × $10^{-2}$	8.63 × $10^{-4}$	8.54 × $10^{-2}$
	Std	3.40 × $10^{-5}$	1.19 × $10^{-4}$	4.13 × $10^{-5}$	3.03 × $10^{-5}$	1.87 × $10^{-4}$	7.05 × $10^{-5}$	3.27 × $10^{-4}$	4.52 × $10^{-2}$	4.98 × $10^{-4}$	4.22 × $10^{-2}$
	Time/s	8.49 × $10^{-1}$	3.63 × $10^{-1}$	6.63 × $10^{-1}$	9.37 × $10^{-1}$	3.87 × $10^{-1}$	5.01 × $10^{-1}$	3.63 × $10^{-1}$	2.78 × $10^{-1}$	3.02 × $10^{-1}$	1.92 × $10^{-1}$
	*p*-value	/	6.34 × $10^{-6}$	3.87 × $10^{-2}$	7.50 × $10^{-1}$	3.52 × $10^{-6}$	6.27 × $10^{-2}$	1.73 × $10^{-6}$	1.73 × $10^{-6}$	1.73 × $10^{-6}$	1.73 × $10^{-6}$
F7	Mean	0.00× $10^{0}$	0.00× $10^{0}$	0.00× $10^{0}$	0.00× $10^{0}$	0.00× $10^{0}$	7.75 × $10^{-6}$	5.56 × $10^{1}$	6.03 × $10^{1}$	3.58 × $10^{-1}$	4.94 × $10^{1}$
	Std	0.00× $10^{0}$	0.00× $10^{0}$	0.00× $10^{0}$	0.00× $10^{0}$	0.00× $10^{0}$	4.24 × $10^{-5}$	8.66 × $10^{1}$	1.43 × $10^{1}$	1.37 × $10^{0}$	9.79 × $10^{0}$
	Time/s	6.05 × $10^{-1}$	2.42 × $10^{-1}$	4.54 × $10^{-1}$	8.26 × $10^{-1}$	2.75 × $10^{-1}$	2.75 × $10^{-1}$	1.68 × $10^{-1}$	1.75 × $10^{-1}$	1.88 × $10^{-1}$	9.09 × $10^{-2}$
	*p*-value	/	1.00 × $10^{0}$	1.00 × $10^{0}$	1.00 × $10^{0}$	1.00 × $10^{0}$	1.00 × $10^{0}$	1.22 × $10^{-4}$	1.73 × $10^{-6}$	3.91 × $10^{-3}$	1.73 × $10^{-6}$
F8	Mean	8.88 × $10^{-16}$	6.65 × $10^{-1}$	4.20 × $10^{-15}$	8.88 × $10^{-16}$	4.56 × $10^{-15}$	8.88 × $10^{-16}$	3.44 × $10^{-11}$	1.92 × $10^{0}$	1.64 × $10^{-14}$	1.05 × $10^{-2}$
	Std	0.00× $10^{0}$	3.64 × $10^{0}$	9.01 × $10^{-16}$	0.00× $10^{0}$	6.49 × $10^{-16}$	0.00× $10^{0}$	1.77 × $10^{-11}$	7.15 × $10^{-1}$	3.55 × $10^{-15}$	1.18 × $10^{-2}$
	Time/s	6.21 × $10^{-1}$	2.51 × $10^{-1}$	4.71 × $10^{-1}$	8.48 × $10^{-1}$	2.77 × $10^{-1}$	2.87 × $10^{-1}$	1.58 × $10^{-1}$	1.86 × $10^{-1}$	1.97 × $10^{-1}$	9.31 × $10^{-2}$
	*p*-value	/	1.00 × $10^{0}$	1.21 × $10^{-7}$	1.00 × $10^{0}$	6.80 × $10^{-8}$	1.00 × $10^{0}$	1.73 × $10^{-6}$	1.73 × $10^{-6}$	3.32 × $10^{-7}$	1.73 × $10^{-6}$
F9	Mean	0.00× $10^{0}$	0.00× $10^{0}$	0.00× $10^{0}$	0.00× $10^{0}$	0.00× $10^{0}$	0.00× $10^{0}$	2.35 × $10^{-15}$	7.96 × $10^{-3}$	2.40 × $10^{-3}$	1.10 × $10^{1}$
	Std	0.00× $10^{0}$	0.00× $10^{0}$	0.00× $10^{0}$	0.00× $10^{0}$	0.00× $10^{0}$	0.00× $10^{0}$	4.91 × $10^{-15}$	1.03 × $10^{-2}$	5.13 × $10^{-3}$	3.91 × $10^{0}$
	Time/s	6.71 × $10^{-1}$	2.73 × $10^{-1}$	5.11 × $10^{-1}$	8.58 × $10^{-1}$	2.99 × $10^{-1}$	3.30 × $10^{-1}$	1.97 × $10^{-1}$	2.07 × $10^{-1}$	2.19 × $10^{-1}$	1.25 × $10^{-1}$
	*p*-value	/	1.00 × $10^{0}$	1.00 × $10^{0}$	1.00 × $10^{0}$	1.00 × $10^{0}$	1.00 × $10^{0}$	2.63 × $10^{-5}$	1.73 × $10^{-6}$	3.13 × $10^{-2}$	1.73 × $10^{-6}$
F10	Mean	7.41 × $10^{-125}$	2.80 × $10^{-92}$	1.20 × $10^{-20}$	2.08 × $10^{-66}$	5.60 × $10^{-90}$	1.07 × $10^{-10}$	2.76 × $10^{-5}$	1.27 × $10^{1}$	1.73 × $10^{-49}$	3.96 × $10^{-2}$
	Std	4.06 × $10^{-124}$	1.24 × $10^{-91}$	6.56 × $10^{-20}$	1.14 × $10^{-65}$	3.07 × $10^{-89}$	5.80 × $10^{-10}$	4.16 × $10^{-5}$	2.69 × $10^{1}$	9.48 × $10^{-49}$	1.11 × $10^{-1}$
	Time/s	8.05 × $10^{-1}$	3.58 × $10^{-1}$	6.51 × $10^{-1}$	9.25 × $10^{-1}$	3.89 × $10^{-1}$	5.00 × $10^{-1}$	3.70 × $10^{-1}$	2.80 × $10^{-1}$	3.08 × $10^{-1}$	1.97 × $10^{-1}$
	*p*-value	/	1.36 × $10^{-5}$	1.24 × $10^{-5}$	1.73 × $10^{-6}$	1.73 × $10^{-6}$	1.73 × $10^{-6}$	1.73 × $10^{-6}$	1.73 × $10^{-6}$	1.73 × $10^{-6}$	1.73 × $10^{-6}$
F11	Mean	5.06 × $10^{3}$	4.99 × $10^{3}$	5.48 × $10^{3}$	9.51 × $10^{3}$	6.73 × $10^{3}$	5.59 × $10^{3}$	8.99 × $10^{3}$	4.95 × $10^{3}$	5.30 × $10^{3}$	5.42 × $10^{3}$
	Std	1.18 × $10^{3}$	9.84 × $10^{2}$	5.34 × $10^{2}$	3.50 × $10^{2}$	1.61 × $10^{3}$	6.53 × $10^{2}$	3.26 × $10^{2}$	7.47 × $10^{2}$	1.73 × $10^{3}$	7.92 × $10^{2}$
	Time/s	7.75 × $10^{-1}$	3.31 × $10^{-1}$	6.93 × $10^{-1}$	9.70 × $10^{-1}$	4.19 × $10^{-1}$	5.60 × $10^{-1}$	4.11 × $10^{-1}$	3.03 × $10^{-1}$	3.32 × $10^{-1}$	2.23 × $10^{-1}$
	*p*-value	/	7.04 × $10^{-1}$	2.11 × $10^{-3}$	1.92 × $10^{-6}$	1.24 × $10^{-5}$	6.04 × $10^{-3}$	1.92 × $10^{-6}$	9.10 × $10^{-1}$	9.10 × $10^{-1}$	7.19 × $10^{-2}$
F12	Mean	3.20 × $10^{4}$	1.91 × $10^{5}$	5.28 × $10^{8}$	1.71 × $10^{10}$	1.86 × $10^{8}$	9.40 × $10^{5}$	8.44 × $10^{9}$	1.25 × $10^{5}$	1.18 × $10^{7}$	2.08 × $10^{4}$
	Std	2.18 × $10^{4}$	8.26 × $10^{5}$	1.07 × $10^{9}$	3.65 × $10^{9}$	2.56 × $10^{8}$	5.88 × $10^{5}$	3.96 × $10^{9}$	6.62 × $10^{4}$	3.66 × $10^{7}$	1.79 × $10^{4}$
	Time/s	7.05 × $10^{-1}$	2.96 × $10^{-1}$	6.41 × $10^{-1}$	9.39 × $10^{-1}$	3.79 × $10^{-1}$	4.85 × $10^{-1}$	3.41 × $10^{-1}$	2.67 × $10^{-1}$	2.93 × $10^{-1}$	1.84 × $10^{-1}$
	*p*-value	/	1.65 × $10^{-1}$	1.73 × $10^{-6}$	1.73 × $10^{-6}$	1.73 × $10^{-6}$	1.73 × $10^{-6}$	1.73 × $10^{-6}$	2.35 × $10^{-6}$	1.92 × $10^{-6}$	9.84 × $10^{-3}$
F13	Mean	1.03 × $10^{4}$	1.12 × $10^{4}$	2.50 × $10^{5}$	9.12 × $10^{8}$	2.62 × $10^{7}$	1.51 × $10^{5}$	5.10 × $10^{8}$	6.98 × $10^{4}$	6.95 × $10^{5}$	9.20 × $10^{3}$
	Std	1.27 × $10^{4}$	9.20 × $10^{3}$	5.34 × $10^{5}$	1.65 × $10^{8}$	8.26 × $10^{7}$	7.03 × $10^{4}$	4.20 × $10^{8}$	4.62 × $10^{4}$	1.28 × $10^{6}$	8.87 × $10^{3}$
	Time/s	6.85 × $10^{-1}$	2.85 × $10^{-1}$	6.25 × $10^{-1}$	9.24 × $10^{-1}$	3.66 × $10^{-1}$	4.67 × $10^{-1}$	3.13 × $10^{-1}$	2.55 × $10^{-1}$	2.86 × $10^{-1}$	1.73 × $10^{-1}$
	*p*-value	/	4.78 × $10^{-1}$	1.48 × $10^{-4}$	1.73 × $10^{-6}$	1.73 × $10^{-6}$	1.73 × $10^{-6}$	1.73 × $10^{-6}$	1.73 × $10^{-6}$	1.73 × $10^{-6}$	7.66 × $10^{-1}$
F14	Mean	1.22 × $10^{4}$	1.09 × $10^{4}$	7.45 × $10^{6}$	1.77 × $10^{9}$	1.42 × $10^{7}$	2.02 × $10^{6}$	4.56 × $10^{8}$	2.98 × $10^{6}$	2.15 × $10^{6}$	9.35 × $10^{3}$
	Std	9.52 × $10^{3}$	1.31 × $10^{4}$	2.32 × $10^{7}$	7.60 × $10^{8}$	3.53 × $10^{7}$	1.80 × $10^{6}$	3.71 × $10^{8}$	1.76 × $10^{6}$	3.08 × $10^{6}$	8.48 × $10^{3}$
	Time/s	1.86 × $10^{0}$	8.75 × $10^{-1}$	1.50 × $10^{0}$	1.52 × $10^{0}$	1.00 × $10^{0}$	1.65 × $10^{0}$	1.49 × $10^{0}$	8.78 × $10^{-1}$	9.20 × $10^{-1}$	7.56 × $10^{-1}$
	*p*-value	/	1.41 × $10^{-1}$	2.60 × $10^{-6}$	1.73 × $10^{-6}$	1.73 × $10^{-6}$	1.73 × $10^{-6}$	1.73 × $10^{-6}$	1.73 × $10^{-6}$	1.92 × $10^{-6}$	2.21 × $10^{-1}$
F15	Mean	3.35 × $10^{3}$	3.39 × $10^{3}$	3.49 × $10^{3}$	5.47 × $10^{3}$	3.36 × $10^{3}$	3.36 × $10^{3}$	4.19 × $10^{3}$	3.26 × $10^{3}$	3.26 × $10^{3}$	3.90 × $10^{3}$
	Std	1.02 × $10^{2}$	1.65 × $10^{2}$	8.39 × $10^{1}$	4.62 × $10^{2}$	7.14 × $10^{1}$	5.18 × $10^{1}$	3.25 × $10^{2}$	2.83 × $10^{1}$	2.61 × $10^{1}$	2.67 × $10^{2}$
	Time/s	1.51 × $10^{0}$	6.94 × $10^{-1}$	1.24 × $10^{0}$	1.34 × $10^{0}$	8.32 × $10^{-1}$	1.30 × $10^{0}$	1.14 × $10^{0}$	6.71 × $10^{-1}$	6.90 × $10^{-1}$	5.76 × $10^{-1}$
	*p*-value	/	2.62 × $10^{-1}$	3.11 × $10^{-5}$	1.73 × $10^{-6}$	7.81 × $10^{-1}$	2.89 × $10^{-1}$	1.73 × $10^{-6}$	8.92 × $10^{-5}$	1.60 × $10^{-4}$	1.92 × $10^{-6}$

**Table 8:** Comparative evaluation of ten algorithms using 15 benchmark functions with Dim = 30.

Function	CQLHBA	HBA	CSHO	SCQSSA	GJO	AO	BOA	SSA	GWO	RPSO
F1	1.00	3.70	2.47	5.00	6.00	2.83	8.00	9.00	7.00	10.00
F2	1.00	3.27	2.00	6.00	5.00	3.73	8.00	9.87	7.00	9.13
F3	1.00	3.53	3.23	5.00	6.00	2.23	7.97	10.00	7.03	9.00
F4	1.00	3.03	3.70	5.00	6.00	2.27	8.00	10.00	7.00	9.00
F5	5.53	1.90	7.77	10.00	7.23	3.13	9.00	1.13	5.47	3.83
F6	2.47	4.97	3.03	2.20	5.37	3.17	7.47	9.57	7.33	9.43
F7	4.10	4.10	4.10	4.10	4.10	4.23	6.63	9.40	5.23	9.00
F8	2.52	2.78	5.28	2.52	5.52	2.52	7.97	9.93	6.97	9.00
F9	4.02	4.02	4.02	4.02	4.02	4.02	6.98	8.87	5.05	10.00
F10	1.07	2.90	3.07	4.93	3.27	6.87	8.20	10.00	5.90	8.80
F11	3.67	3.67	5.33	9.77	6.67	5.33	9.00	3.60	3.33	4.63
F12	2.17	2.73	7.40	9.93	7.27	5.93	9.07	4.37	4.60	1.53
F13	2.03	2.40	4.40	9.83	7.10	6.57	9.13	5.50	6.07	1.97
F14	2.43	1.80	4.97	9.97	6.30	6.13	9.00	6.87	5.60	1.93
F15	4.03	4.57	6.47	10.00	4.30	4.73	8.70	1.97	2.03	8.20
Sum	38.03	49.37	67.23	98.27	84.13	63.70	123.12	110.07	85.62	105.47
Mean	2.54	3.29	4.48	6.55	5.61	4.25	8.21	7.34	5.71	7.03

**Table 9:** Comparative evaluation of ten algorithms using 15 benchmark functions with Dim = 100.

Function	CQLHBA	HBA	CSHO	SCQSSA	GJO	AO	BOA	SSA	GWO	RPSO
F1	1.00	2.97	3.57	5.00	6.00	2.47	8.00	9.50	7.00	9.50
F2	1.00	3.43	3.17	5.00	6.00	2.40	10.00	9.00	7.00	8.00
F3	1.00	3.97	3.03	5.00	6.70	2.00	6.30	10.00	8.00	9.00
F4	1.00	4.00	3.00	5.00	7.43	2.00	6.00	10.00	7.63	8.93
F5	5.93	3.80	7.90	10.00	7.10	1.00	9.00	2.40	5.07	2.80
F6	2.97	4.77	2.67	2.13	6.07	2.77	6.67	9.00	7.97	10.00
F7	4.07	4.07	4.07	4.07	4.07	4.07	4.20	9.00	7.40	10.00
F8	2.43	3.77	4.77	2.43	5.83	2.43	7.83	9.83	6.83	8.83
F9	4.00	4.00	4.00	4.00	4.00	4.00	8.00	9.00	4.00	10.00
F10	1.00	2.63	3.03	4.80	3.67	6.87	10.00	8.23	6.00	8.77
F11	3.30	4.33	7.00	9.87	6.57	6.37	9.07	2.30	2.70	3.50
F12	2.60	2.67	7.97	9.83	7.03	5.13	9.17	3.47	5.87	1.27
F13	1.73	2.50	7.90	9.93	7.07	5.20	9.07	3.93	5.83	1.83
F14	1.57	1.53	7.87	9.97	7.07	4.90	9.03	4.30	5.87	2.90
F15	3.17	2.47	6.97	10.00	5.03	5.70	9.00	2.20	3.10	7.37
Sum	36.77	50.90	76.90	97.03	89.63	57.30	121.33	102.17	90.27	102.70
Mean	2.45	3.39	5.13	6.47	5.98	3.82	8.09	6.81	6.02	6.85

**Table 10:** Results of ten algorithms using 15 benchmark functions with Dim = 100.

Function	Item	CQLHBA	HBA	CSHO	SCQSSA	GJO	AO	BOA	SSA	GWO	RPSO
F1	Mean	0.00× $10^{0}$	1.02 × $10^{-250}$	3.75 × $10^{-246}$	2.83 × $10^{-119}$	4.49 × $10^{-60}$	3.02 × $10^{-197}$	1.26 × $10^{-13}$	3.14 × $10^{0}$	1.54 × $10^{-29}$	2.59 × $10^{0}$
	Std	0.00× $10^{0}$	0.00× $10^{0}$	0.00× $10^{0}$	1.38 × $10^{-118}$	8.65 × $10^{-60}$	0.00× $10^{0}$	9.70 × $10^{-15}$	1.84 × $10^{0}$	1.62 × $10^{-29}$	7.24 × $10^{-1}$
	Time/s	9.59 × $10^{-1}$	4.25 × $10^{-1}$	1.13 × $10^{0}$	2.58 × $10^{0}$	5.25 × $10^{-1}$	4.37 × $10^{-1}$	1.59 × $10^{-1}$	3.75 × $10^{-1}$	5.10 × $10^{-1}$	1.92 × $10^{-1}$
	*p*-value	/	1.73 × $10^{-6}$	1.73 × $10^{-6}$	1.73 × $10^{-6}$	1.73 × $10^{-6}$	1.73 × $10^{-6}$	1.73 × $10^{-6}$	1.73 × $10^{-6}$	1.73 × $10^{-6}$	1.73 × $10^{-6}$
F2	Mean	0.00× $10^{0}$	9.00 × $10^{-134}$	3.68 × $10^{-134}$	4.23 × $10^{-48}$	3.92 × $10^{-37}$	1.34 × $10^{-113}$	1.76 × $10^{50}$	2.37 × $10^{1}$	6.12 × $10^{-18}$	9.73 × $10^{0}$
	Std	0.00× $10^{0}$	1.71 × $10^{-133}$	1.01 × $10^{-133}$	8.66 × $10^{-48}$	5.85 × $10^{-37}$	7.35 × $10^{-113}$	6.62 × $10^{50}$	5.37 × $10^{0}$	3.42 × $10^{-18}$	1.73 × $10^{0}$
	Time/s	9.88 × $10^{-1}$	4.40 × $10^{-1}$	1.13 × $10^{0}$	2.57 × $10^{0}$	5.39 × $10^{-1}$	4.50 × $10^{-1}$	1.63 × $10^{-1}$	3.77 × $10^{-1}$	5.09 × $10^{-1}$	1.92 × $10^{-1}$
	*p*-value	/	1.73 × $10^{-6}$	1.73 × $10^{-6}$	1.73 × $10^{-6}$	1.73 × $10^{-6}$	1.73 × $10^{-6}$	1.73 × $10^{-6}$	1.73 × $10^{-6}$	1.73 × $10^{-6}$	1.73 × $10^{-6}$
F3	Mean	0.00× $10^{0}$	5.95 × $10^{-169}$	1.46 × $10^{-177}$	3.55 × $10^{-119}$	6.56 × $10^{-5}$	6.21 × $10^{-195}$	1.05 × $10^{-13}$	3.15 × $10^{4}$	5.94 × $10^{0}$	9.44 × $10^{3}$
	Std	0.00× $10^{0}$	0.00× $10^{0}$	0.00× $10^{0}$	1.33 × $10^{-118}$	3.45 × $10^{-4}$	0.00× $10^{0}$	6.52 × $10^{-15}$	1.49 × $10^{4}$	1.21 × $10^{1}$	3.07 × $10^{3}$
	Time/s	2.97 × $10^{0}$	1.37 × $10^{0}$	2.54 × $10^{0}$	3.50 × $10^{0}$	1.45 × $10^{0}$	2.30 × $10^{0}$	2.02 × $10^{0}$	1.30 × $10^{0}$	1.43 × $10^{0}$	1.11 × $10^{0}$
	*p*-value	/	1.73 × $10^{-6}$	1.73 × $10^{-6}$	1.73 × $10^{-6}$	1.73 × $10^{-6}$	1.73 × $10^{-6}$	1.73 × $10^{-6}$	1.73 × $10^{-6}$	1.73 × $10^{-6}$	1.73 × $10^{-6}$
F4	Mean	0.00× $10^{0}$	8.41 × $10^{-81}$	1.49 × $10^{-102}$	1.35 × $10^{-60}$	1.34 × $10^{0}$	1.57 × $10^{-105}$	8.11 × $10^{-11}$	2.64 × $10^{1}$	4.03 × $10^{-3}$	8.67 × $10^{0}$
	Std	0.00× $10^{0}$	4.20 × $10^{-80}$	5.84 × $10^{-102}$	4.00 × $10^{-60}$	3.86 × $10^{0}$	8.18 × $10^{-105}$	5.11 × $10^{-12}$	3.73 × $10^{0}$	6.96 × $10^{-3}$	1.10 × $10^{0}$
	Time/s	9.62 × $10^{-1}$	4.25 × $10^{-1}$	1.12 × $10^{0}$	2.59 × $10^{0}$	5.20 × $10^{-1}$	4.33 × $10^{-1}$	1.55 × $10^{-1}$	3.67 × $10^{-1}$	5.00 × $10^{-1}$	1.91 × $10^{-1}$
	*p*-value	/	1.73 × $10^{-6}$	1.73 × $10^{-6}$	1.73 × $10^{-6}$	1.73 × $10^{-6}$	1.73 × $10^{-6}$	1.73 × $10^{-6}$	1.73 × $10^{-6}$	1.73 × $10^{-6}$	1.73 × $10^{-6}$
F5	Mean	1.13 × $10^{1}$	4.34 × $10^{0}$	1.83 × $10^{1}$	2.50 × $10^{1}$	1.66 × $10^{1}$	9.34 × $10^{-5}$	2.26 × $10^{1}$	2.29 × $10^{0}$	9.40 × $10^{0}$	2.71 × $10^{0}$
	Std	1.02 × $10^{0}$	8.47 × $10^{-1}$	8.63 × $10^{-1}$	0.00× $10^{0}$	8.94 × $10^{-1}$	1.44 × $10^{-4}$	7.01 × $10^{-1}$	1.48 × $10^{0}$	7.33 × $10^{-1}$	8.60 × $10^{-1}$
	Time/s	9.47 × $10^{-1}$	4.40 × $10^{-1}$	1.12 × $10^{0}$	2.58 × $10^{0}$	5.23 × $10^{-1}$	4.33 × $10^{-1}$	1.52 × $10^{-1}$	3.67 × $10^{-1}$	5.08 × $10^{-1}$	1.92 × $10^{-1}$
	*p*-value	/	1.73 × $10^{-6}$	1.73 × $10^{-6}$	1.73 × $10^{-6}$	1.73 × $10^{-6}$	1.73 × $10^{-6}$	1.73 × $10^{-6}$	1.73 × $10^{-6}$	2.60 × $10^{-6}$	1.73 × $10^{-6}$
F6	Mean	4.79 × $10^{-5}$	2.57 × $10^{-4}$	4.78 × $10^{-5}$	4.01 × $10^{-5}$	5.88 × $10^{-4}$	5.19 × $10^{-5}$	9.22 × $10^{-4}$	1.32 × $10^{0}$	2.88 × $10^{-3}$	2.55 × $10^{1}$
	Std	3.40 × $10^{-5}$	2.90 × $10^{-4}$	3.54 × $10^{-5}$	4.38 × $10^{-5}$	3.76 × $10^{-4}$	5.40 × $10^{-5}$	4.14 × $10^{-4}$	3.46 × $10^{-1}$	1.43 × $10^{-3}$	1.03 × $10^{1}$
	Time/s	1.69 × $10^{0}$	7.95 × $10^{-1}$	1.68 × $10^{0}$	2.91 × $10^{0}$	9.03 × $10^{-1}$	1.19 × $10^{0}$	9.04 × $10^{-1}$	7.19 × $10^{-1}$	8.72 × $10^{-1}$	5.56 × $10^{-1}$
	*p*-value	/	3.11 × $10^{-5}$	6.29 × $10^{-1}$	1.99 × $10^{-1}$	1.73 × $10^{-6}$	7.97 × $10^{-1}$	1.73 × $10^{-6}$	1.73 × $10^{-6}$	1.73 × $10^{-6}$	1.73 × $10^{-6}$
F7	Mean	0.00× $10^{0}$	0.00× $10^{0}$	0.00× $10^{0}$	0.00× $10^{0}$	0.00× $10^{0}$	0.00× $10^{0}$	2.11 × $10^{-11}$	1.63 × $10^{2}$	2.45 × $10^{-1}$	3.66 × $10^{2}$
	Std	0.00× $10^{0}$	0.00× $10^{0}$	0.00× $10^{0}$	0.00× $10^{0}$	0.00× $10^{0}$	0.00× $10^{0}$	1.15 × $10^{-10}$	4.03 × $10^{1}$	1.34 × $10^{0}$	3.20 × $10^{1}$
	Time/s	9.64 × $10^{-1}$	4.58 × $10^{-1}$	1.14 × $10^{0}$	2.55 × $10^{0}$	5.39 × $10^{-1}$	4.98 × $10^{-1}$	2.94 × $10^{-1}$	4.00 × $10^{-1}$	5.17 × $10^{-1}$	2.56 × $10^{-1}$
	*p*-value	/	1.00 × $10^{0}$	1.00 × $10^{0}$	1.00 × $10^{0}$	1.00 × $10^{0}$	1.00 × $10^{0}$	1.00 × $10^{0}$	1.73 × $10^{-6}$	8.93 × $10^{-6}$	1.73 × $10^{-6}$
F8	Mean	8.88 × $10^{-16}$	3.32 × $10^{0}$	4.32 × $10^{-15}$	8.88 × $10^{-16}$	9.30 × $10^{-15}$	8.88 × $10^{-16}$	7.44 × $10^{-11}$	6.69 × $10^{0}$	1.13 × $10^{-13}$	1.92 × $10^{0}$
	Std	0.00× $10^{0}$	7.55 × $10^{0}$	6.49 × $10^{-16}$	0.00× $10^{0}$	2.55 × $10^{-15}$	0.00× $10^{0}$	6.11 × $10^{-12}$	8.80 × $10^{-1}$	1.00 × $10^{-14}$	2.97 × $10^{-1}$
	Time/s	9.82 × $10^{-1}$	4.79 × $10^{-1}$	1.15 × $10^{0}$	2.60 × $10^{0}$	5.47 × $10^{-1}$	5.11 × $10^{-1}$	2.23 × $10^{-1}$	4.28 × $10^{-1}$	5.27 × $10^{-1}$	2.44 × $10^{-1}$
	*p*-value	/	6.25 × $10^{-2}$	7.24 × $10^{-8}$	1.00 × $10^{0}$	4.13 × $10^{-7}$	1.00 × $10^{0}$	1.73 × $10^{-6}$	1.73 × $10^{-6}$	1.52 × $10^{-6}$	1.73 × $10^{-6}$
F9	Mean	0.00× $10^{0}$	0.00× $10^{0}$	0.00× $10^{0}$	0.00× $10^{0}$	0.00× $10^{0}$	0.00× $10^{0}$	7.16 × $10^{-14}$	7.11 × $10^{-1}$	0.00× $10^{0}$	5.27 × $10^{1}$
	Std	0.00× $10^{0}$	0.00× $10^{0}$	0.00× $10^{0}$	0.00× $10^{0}$	0.00× $10^{0}$	0.00× $10^{0}$	5.59 × $10^{-14}$	2.03 × $10^{-1}$	0.00× $10^{0}$	7.73 × $10^{0}$
	Time/s	1.06 × $10^{0}$	4.98 × $10^{-1}$	1.22 × $10^{0}$	2.63 × $10^{0}$	5.91 × $10^{-1}$	5.73 × $10^{-1}$	2.99 × $10^{-1}$	4.51 × $10^{-1}$	5.63 × $10^{-1}$	3.08 × $10^{-1}$
	*p*-value	/	1.00 × $10^{0}$	1.00 × $10^{0}$	1.00 × $10^{0}$	1.00 × $10^{0}$	1.00 × $10^{0}$	1.73 × $10^{-6}$	1.73 × $10^{-6}$	1.00 × $10^{0}$	1.73 × $10^{-6}$
F10	Mean	2.94 × $10^{-187}$	8.67 × $10^{-95}$	2.90 × $10^{-45}$	5.03 × $10^{-71}$	4.06 × $10^{-62}$	2.33 × $10^{-11}$	1.47 × $10^{57}$	1.61 × $10^{13}$	6.73 × $10^{-26}$	1.83 × $10^{12}$
	Std	0.00× $10^{0}$	4.71 × $10^{-94}$	1.59 × $10^{-44}$	1.87 × $10^{-70}$	2.22 × $10^{-61}$	9.08 × $10^{-11}$	5.68 × $10^{57}$	8.64 × $10^{13}$	3.69 × $10^{-25}$	4.88 × $10^{12}$
	Time/s	1.61 × $10^{0}$	8.11 × $10^{-1}$	1.65 × $10^{0}$	2.91 × $10^{0}$	9.20 × $10^{-1}$	1.26 × $10^{0}$	1.03 × $10^{0}$	7.83 × $10^{-1}$	9.26 × $10^{-1}$	6.17 × $10^{-1}$
	*p*-value	/	1.73 × $10^{-6}$	1.73 × $10^{-6}$	1.73 × $10^{-6}$	1.73 × $10^{-6}$	1.73 × $10^{-6}$	1.73 × $10^{-6}$	1.73 × $10^{-6}$	1.73 × $10^{-6}$	1.73 × $10^{-6}$
F11	Mean	5.06 × $10^{3}$	4.99 × $10^{3}$	5.48 × $10^{3}$	9.51 × $10^{3}$	6.73 × $10^{3}$	5.59 × $10^{3}$	8.99 × $10^{3}$	4.95 × $10^{3}$	5.30 × $10^{3}$	5.42 × $10^{3}$
	Std	1.18 × $10^{3}$	9.84 × $10^{2}$	5.34 × $10^{2}$	3.50 × $10^{2}$	1.61 × $10^{3}$	6.53 × $10^{2}$	3.26 × $10^{2}$	7.47 × $10^{2}$	1.73 × $10^{3}$	7.92 × $10^{2}$
	Time/s	7.75 × $10^{-1}$	3.31 × $10^{-1}$	6.93 × $10^{-1}$	9.70 × $10^{-1}$	4.19 × $10^{-1}$	5.60 × $10^{-1}$	4.11 × $10^{-1}$	3.03 × $10^{-1}$	3.32 × $10^{-1}$	2.23 × $10^{-1}$
	*p*-value	/	4.72 × $10^{-2}$	1.92 × $10^{-6}$	1.73 × $10^{-6}$	3.52 × $10^{-6}$	1.73 × $10^{-6}$	1.73 × $10^{-6}$	3.68 × $10^{-2}$	1.78 × $10^{-1}$	5.86 × $10^{-1}$
F12	Mean	3.20 × $10^{4}$	1.91 × $10^{5}$	5.28 × $10^{8}$	1.71 × $10^{10}$	1.86 × $10^{8}$	9.40 × $10^{5}$	8.44 × $10^{9}$	1.25 × $10^{5}$	1.18 × $10^{7}$	2.08 × $10^{4}$
	Std	2.18 × $10^{4}$	8.26 × $10^{5}$	1.07 × $10^{9}$	3.65 × $10^{9}$	2.56 × $10^{8}$	5.88 × $10^{5}$	3.96 × $10^{9}$	6.62 × $10^{4}$	3.66 × $10^{7}$	1.79 × $10^{4}$
	Time/s	7.05 × $10^{-1}$	2.96 × $10^{-1}$	6.41 × $10^{-1}$	9.39 × $10^{-1}$	3.79 × $10^{-1}$	4.85 × $10^{-1}$	3.41 × $10^{-1}$	2.67 × $10^{-1}$	2.93 × $10^{-1}$	1.84 × $10^{-1}$
	*p*-value	/	9.59 × $10^{-1}$	1.73 × $10^{-6}$	1.73 × $10^{-6}$	1.73 × $10^{-6}$	1.73 × $10^{-6}$	1.73 × $10^{-6}$	1.85 × $10^{-2}$	1.73 × $10^{-6}$	1.02 × $10^{-5}$
F13	Mean	1.03 × $10^{4}$	1.12 × $10^{4}$	2.50 × $10^{5}$	9.12 × $10^{8}$	2.62 × $10^{7}$	1.51 × $10^{5}$	5.10 × $10^{8}$	6.98 × $10^{4}$	6.95 × $10^{5}$	9.20 × $10^{3}$
	Std	1.27 × $10^{4}$	9.20 × $10^{3}$	5.34 × $10^{5}$	1.65 × $10^{8}$	8.26 × $10^{7}$	7.03 × $10^{4}$	4.20 × $10^{8}$	4.62 × $10^{4}$	1.28 × $10^{6}$	8.87 × $10^{3}$
	Time/s	6.85 × $10^{-1}$	2.85 × $10^{-1}$	6.25 × $10^{-1}$	9.24 × $10^{-1}$	3.66 × $10^{-1}$	4.67 × $10^{-1}$	3.13 × $10^{-1}$	2.55 × $10^{-1}$	2.86 × $10^{-1}$	1.73 × $10^{-1}$
	*p*-value	/	1.75 × $10^{-2}$	1.73 × $10^{-6}$	1.73 × $10^{-6}$	1.73 × $10^{-6}$	1.73 × $10^{-6}$	1.73 × $10^{-6}$	2.13 × $10^{-6}$	1.73 × $10^{-6}$	3.18 × $10^{-1}$
F14	Mean	1.22 × $10^{4}$	1.09 × $10^{4}$	7.45 × $10^{6}$	1.77 × $10^{9}$	1.42 × $10^{7}$	2.02 × $10^{6}$	4.56 × $10^{8}$	2.98 × $10^{6}$	2.15 × $10^{6}$	9.35 × $10^{3}$
	Std	9.52 × $10^{3}$	1.31 × $10^{4}$	2.32 × $10^{7}$	7.60 × $10^{8}$	3.53 × $10^{7}$	1.80 × $10^{6}$	3.71 × $10^{8}$	1.76 × $10^{6}$	3.08 × $10^{6}$	8.48 × $10^{3}$
	Time/s	1.86 × $10^{0}$	8.75 × $10^{-1}$	1.50 × $10^{0}$	1.52 × $10^{0}$	1.00 × $10^{0}$	1.65 × $10^{0}$	1.49 × $10^{0}$	8.78 × $10^{-1}$	9.20 × $10^{-1}$	7.56 × $10^{-1}$
	*p*-value	/	4.65 × $10^{-1}$	1.73 × $10^{-6}$	1.73 × $10^{-6}$	1.73 × $10^{-6}$	1.73 × $10^{-6}$	1.73 × $10^{-6}$	1.73 × $10^{-6}$	1.73 × $10^{-6}$	2.88 × $10^{-6}$
F15	Mean	3.35 × $10^{3}$	3.39 × $10^{3}$	3.49 × $10^{3}$	5.47 × $10^{3}$	3.36 × $10^{3}$	3.36 × $10^{3}$	4.19 × $10^{3}$	3.26 × $10^{3}$	3.26 × $10^{3}$	3.90 × $10^{3}$
	Std	1.02 × $10^{2}$	1.65 × $10^{2}$	8.39 × $10^{1}$	4.62 × $10^{2}$	7.14 × $10^{1}$	5.18 × $10^{1}$	3.25 × $10^{2}$	2.83 × $10^{1}$	2.61 × $10^{1}$	2.67 × $10^{2}$
	Time/s	1.51 × $10^{0}$	6.94 × $10^{-1}$	1.24 × $10^{0}$	1.34 × $10^{0}$	8.32 × $10^{-1}$	1.30 × $10^{0}$	1.14 × $10^{0}$	6.71 × $10^{-1}$	6.90 × $10^{-1}$	5.76 × $10^{-1}$
	*p*-value	/	1.99 × $10^{-1}$	4.07 × $10^{-5}$	1.73 × $10^{-6}$	1.11 × $10^{-3}$	1.25 × $10^{-4}$	1.73 × $10^{-6}$	4.72 × $10^{-2}$	5.30 × $10^{-1}$	2.60 × $10^{-5}$

**Table 11:** Results of ten algorithms using six test functions from CEC2022 with Dim = 20.

Function	Item	CQLHBA	HBA	CSHO	SCQSSA	GJO	AO	BOA	SSA	GWO	RPSO
F16	Mean	4.03 × $10^{2}$	6.69 × $10^{2}$	1.66 × $10^{4}$	1.86 × $10^{8}$	1.50 × $10^{4}$	4.62 × $10^{4}$	6.33 × $10^{4}$	8.29 × $10^{2}$	1.28 × $10^{4}$	3.90 × $10^{2}$
	Std	1.56 × $10^{2}$	3.39 × $10^{2}$	3.98 × $10^{3}$	3.81 × $10^{8}$	4.80 × $10^{3}$	1.22 × $10^{4}$	2.35 × $10^{4}$	6.68 × $10^{2}$	3.50 × $10^{3}$	1.30 × $10^{2}$
	Time/s	5.13 × $10^{-1}$	2.01 × $10^{-1}$	4.27 × $10^{-1}$	6.69 × $10^{-1}$	2.91 × $10^{-1}$	3.53 × $10^{-1}$	2.34 × $10^{-1}$	2.08 × $10^{-1}$	1.98 × $10^{-1}$	1.10 × $10^{-1}$
	*p*-value	/	8.31 × $10^{-4}$	1.73 × $10^{-6}$	1.73 × $10^{-6}$	1.73 × $10^{-6}$	1.73 × $10^{-6}$	1.73 × $10^{-6}$	1.29 × $10^{-3}$	1.73 × $10^{-6}$	6.73 × $10^{-1}$
F17	Mean	4.53 × $10^{2}$	4.56 × $10^{2}$	7.50 × $10^{2}$	4.19 × $10^{3}$	6.15 × $10^{2}$	5.22 × $10^{2}$	3.19 × $10^{3}$	4.59 × $10^{2}$	5.03 × $10^{2}$	4.39 × $10^{2}$
	Std	2.17 × $10^{1}$	1.43 × $10^{1}$	1.86 × $10^{2}$	6.33 × $10^{2}$	1.06 × $10^{2}$	4.45 × $10^{1}$	8.85 × $10^{2}$	2.34 × $10^{1}$	3.59 × $10^{1}$	2.80 × $10^{1}$
	Time/s	5.19 × $10^{-1}$	2.06 × $10^{-1}$	4.29 × $10^{-1}$	6.87 × $10^{-1}$	2.97 × $10^{-1}$	3.57 × $10^{-1}$	2.28 × $10^{-1}$	2.07 × $10^{-1}$	2.03 × $10^{-1}$	1.09 × $10^{-1}$
	*p*-value	/	9.75 × $10^{-1}$	1.73 × $10^{-6}$	1.73 × $10^{-6}$	1.73 × $10^{-6}$	1.92 × $10^{-6}$	1.73 × $10^{-6}$	8.77 × $10^{-1}$	6.34 × $10^{-6}$	7.86 × $10^{-2}$
F18	Mean	7.14 × $10^{3}$	9.36 × $10^{3}$	8.84 × $10^{6}$	4.87 × $10^{9}$	3.79 × $10^{7}$	1.56 × $10^{5}$	1.74 × $10^{9}$	8.37 × $10^{3}$	2.54 × $10^{6}$	4.45 × $10^{3}$
	Std	7.06 × $10^{3}$	7.49 × $10^{3}$	1.33 × $10^{7}$	1.35 × $10^{9}$	5.41 × $10^{7}$	8.08 × $10^{4}$	1.06 × $10^{9}$	7.02 × $10^{3}$	6.72 × $10^{6}$	3.55 × $10^{3}$
	Time/s	5.32 × $10^{-1}$	2.08 × $10^{-1}$	4.39 × $10^{-1}$	6.74 × $10^{-1}$	3.00 × $10^{-1}$	3.61 × $10^{-1}$	2.34 × $10^{-1}$	2.10 × $10^{-1}$	2.05 × $10^{-1}$	1.15 × $10^{-1}$
	*p*-value	/	1.85 × $10^{-1}$	1.73 × $10^{-6}$	1.73 × $10^{-6}$	1.73 × $10^{-6}$	1.73 × $10^{-6}$	1.73 × $10^{-6}$	2.54 × $10^{-1}$	5.75 × $10^{-6}$	1.59 × $10^{-1}$
F19	Mean	2.09 × $10^{3}$	2.11 × $10^{3}$	2.11 × $10^{3}$	2.41 × $10^{3}$	2.12 × $10^{3}$	2.11 × $10^{3}$	2.19 × $10^{3}$	2.11 × $10^{3}$	2.09 × $10^{3}$	2.14 × $10^{3}$
	Std	5.57 × $10^{1}$	7.64 × $10^{1}$	2.91 × $10^{1}$	7.89 × $10^{1}$	4.90 × $10^{1}$	3.29 × $10^{1}$	2.28 × $10^{1}$	3.98 × $10^{1}$	5.58 × $10^{1}$	5.25 × $10^{1}$
	Time/s	8.28 × $10^{-1}$	3.47 × $10^{-1}$	6.64 × $10^{-1}$	8.08 × $10^{-1}$	4.40 × $10^{-1}$	6.39 × $10^{-1}$	5.09 × $10^{-1}$	3.48 × $10^{-1}$	3.40 × $10^{-1}$	2.54 × $10^{-1}$
	*p*-value	/	4.91 × $10^{-1}$	1.02 × $10^{-1}$	1.73 × $10^{-6}$	8.22 × $10^{-3}$	6.87 × $10^{-2}$	7.69 × $10^{-6}$	1.06 × $10^{-1}$	9.43 × $10^{-1}$	3.61 × $10^{-3}$
F20	Mean	2.48 × $10^{3}$	2.48 × $10^{3}$	2.60 × $10^{3}$	4.10 × $10^{3}$	2.58 × $10^{3}$	2.56 × $10^{3}$	3.98 × $10^{3}$	2.50 × $10^{3}$	2.52 × $10^{3}$	2.47 × $10^{3}$
	Std	3.78 × $10^{0}$	5.24 × $10^{-2}$	5.41 × $10^{1}$	3.43 × $10^{2}$	5.34 × $10^{1}$	4.29 × $10^{1}$	4.74 × $10^{2}$	2.70 × $10^{1}$	3.23 × $10^{1}$	5.66 × $10^{0}$
	Time/s	8.31 × $10^{-1}$	5.16 × $10^{-1}$	1.12 × $10^{0}$	9.04 × $10^{-1}$	4.75 × $10^{-1}$	6.29 × $10^{-1}$	5.00 × $10^{-1}$	3.45 × $10^{-1}$	3.32 × $10^{-1}$	2.41 × $10^{-1}$
	*p*-value	/	1.65 × $10^{-1}$	1.73 × $10^{-6}$	1.73 × $10^{-6}$	1.73 × $10^{-6}$	1.73 × $10^{-6}$	1.73 × $10^{-6}$	1.36 × $10^{-5}$	1.73 × $10^{-6}$	2.16 × $10^{-5}$
F21	Mean	3.88 × $10^{3}$	3.61 × $10^{3}$	3.27 × $10^{3}$	7.11 × $10^{3}$	4.03 × $10^{3}$	3.41 × $10^{3}$	3.88 × $10^{3}$	4.09 × $10^{3}$	3.77 × $10^{3}$	4.38 × $10^{3}$
	Std	9.86 × $10^{2}$	1.18 × $10^{3}$	6.01 × $10^{2}$	1.24 × $10^{3}$	1.47 × $10^{3}$	1.16 × $10^{3}$	1.85 × $10^{3}$	1.29 × $10^{3}$	8.93 × $10^{2}$	9.95 × $10^{2}$
	Time/s	7.22 × $10^{-1}$	2.98 × $10^{-1}$	5.83 × $10^{-1}$	7.72 × $10^{-1}$	3.86 × $10^{-1}$	5.37 × $10^{-1}$	4.09 × $10^{-1}$	2.97 × $10^{-1}$	2.86 × $10^{-1}$	1.98 × $10^{-1}$
	*p*-value	/	2.99 × $10^{-1}$	2.18 × $10^{-2}$	1.73 × $10^{-6}$	5.04 × $10^{-1}$	2.62 × $10^{-1}$	4.91 × $10^{-1}$	3.82 × $10^{-1}$	9.92 × $10^{-1}$	5.19 × $10^{-2}$

**Table 12:** Comparative evaluation of ten algorithms using six test functions from CEC2022 with Dim = 20.

Function	CQLHBA	HBA	CSHO	SCQSSA	GJO	AO	BOA	SSA	GWO	RPSO
F16	2.00	3.10	6.47	9.83	5.93	8.23	8.93	3.03	5.60	1.87
F17	2.87	2.57	7.77	9.87	6.93	5.87	9.13	2.67	5.00	2.33
F18	2.33	3.20	6.83	9.97	7.50	6.00	9.03	2.90	5.20	2.03
F19	3.43	3.70	5.00	10.00	5.30	4.93	8.40	4.50	3.67	6.07
F20	2.63	2.33	7.20	9.67	6.93	6.33	9.33	4.40	5.03	1.13
F21	4.97	4.60	4.13	9.57	5.50	4.30	5.00	5.67	4.90	6.37
Sum	18.23	19.50	37.40	58.90	38.10	35.67	49.83	23.17	29.40	19.80
Mean	3.04	3.25	6.23	9.82	6.35	5.94	8.31	3.86	4.90	3.30

**Table 13:** Results of the comparison methods for the TBTD problem.

Methods	$x_{1}\left(A_{1}\right)$	$x_{2}\left(A_{2}\right)$	$f_{\min}$
PSO	0.7286	0.6460	270.6948
GWO	0.7889	0.4076	263.8970
AO	0.7887	0.4085	263.9236
BOA	0.7720	0.4614	264.5075
GJO	0.7826	0.4258	263.9302
CSHO	0.7987	0.3806	263.9674
HBA	0.7881	0.4099	263.8961
CQLHBA	0.7887	0.4083	263.8961

**Table 14:** Results of the comparison methods for the TSD problem.

Methods	$x_{1}\left(d\right)$	$x_{2}\left(D\right)$	$x_{3}\left(N\right)$	$f_{\min}$
PSO	0.0701	0.9605	2.0000	0.0188
L-SHADE	0.0839	0.9342	4.5046	0.0428
WOA	0.0523	0.3720	10.4470	0.0127
BOA	0.0500	0.3148	14.5890	0.0131
GJO	0.0500	0.3169	14.1009	0.0128
CSHO	0.0500	0.3165	14.1555	0.0129
HBA	0.0541	0.4186	8.4081	0.0128
CQLHBA	0.0500	0.3174	14.0311	0.0127

**Table 15:** Comparison of algorithms on the NCO problem with a changing sensing radius.

Methods	R = 15, Nodes = 20		R = 10, Nodes = 45	
	Coverage	Time/s	Coverage	Time/s
CQLHBA (our)	97.57%	2.41	95.94%	4.61
HBA	94.28%	2.10	90.23%	3.91
DSA	94.38%	5.24	92.37%	9.74
GJO	87.02%	3.98	84.18%	7.55
MPA	91.53%	4.45	90.13%	8.38
GWO	89.53%	5.39	84.04%	10.54
RPSO	94.13%	2.57	90.16%	4.24
BOA	89.60%	2.63	84.97%	4.87

## Data Availability

All required data are described in the article.

## Abbreviations

The following abbreviations are used in this manuscript:

PSO	Particle Swarm Optimization
RPSO	Randomised Particle Swarm Optimizer
GWO	Grey Wolf Optimizer
BOA	Butterfly Optimization Algorithm
SSA	Salp Swarm Algorithm
AO	Aquila Optimizer
HBA	Honey Badger Algorithm
DSA	Duck Swarm Algorithm
GJO	Golden Jackal Optimization
SCQSSA	Sine-Cosine Quantum Salp Swarm Algorithm
CSHO	Chaotic Sea-Horse Optimizer
WSN	Wireless Sensor Network
